# Metabolic classification suggests the GLUT1/ALDOB/G6PD axis as a therapeutic target in chemotherapy-resistant pancreatic cancer

**DOI:** 10.1016/j.xcrm.2023.101162

**Published:** 2023-08-18

**Authors:** Yunguang Li, Shijie Tang, Xiaohan Shi, Jingwen Lv, Xueyuan Wu, Yehan Zhang, Huan Wang, Juan He, Yiqin Zhu, Yi Ju, Yajuan Zhang, Shiwei Guo, Weiwei Yang, Huiyong Yin, Luonan Chen, Dong Gao, Gang Jin

**Affiliations:** 1 State Key Laboratory of Cell Biology, Shanghai Key Laboratory of Molecular Andrology, Shanghai Institute of Biochemistry and Cell Biology, Center for Excellence in Molecular Cell Science, Chinese Academy of Sciences, Shanghai 200031, China; 2 University of Chinese Academy of Sciences, Beijing 100049, China; 3 Department of Hepatobiliary Pancreatic Surgery, Changhai Hospital, Second Military Medical University (Naval Medical University), Shanghai 200433, China; 4 CAS Key Laboratory of Nutrition, Metabolism and Food Safety Research, Shanghai Institute of Nutrition and Health (SINH), Innovation Center for Intervention of Chronic Disease and Promotion of Health, Chinese Academy of Sciences (CAS), Shanghai 200031, China; 5 Key Laboratory of Systems Health Science of Zhejiang Province, School of Life Science, Hangzhou Institute for Advanced Study, University of Chinese Academy of Sciences, Chinese Academy of Sciences, Hangzhou 310024, China; 6 School of Life Science and Technology, ShanghaiTech University, Shanghai 201210, China; 7 Department of Biomedical Sciences, City University of Hong Kong, Hong Kong SAR, China

## Abstract

Metabolic reprogramming is known as an emerging mechanism of chemotherapy resistance, but the metabolic signatures of pancreatic ductal adenocarcinomas (PDACs) remain unclear. Here, we characterize the metabolomic profile of PDAC organoids and classify them into glucomet-PDAC (high glucose metabolism levels) and lipomet-PDAC (high lipid metabolism levels). Glucomet-PDACs are more resistant to chemotherapy than lipomet-PDACs, and patients with glucomet-PDAC have a worse prognosis. Integrated analyses reveal that the GLUT1/aldolase B (ALDOB)/glucose-6-phosphate dehydrogenase (G6PD) axis induces chemotherapy resistance by remodeling glucose metabolism in glucomet-PDAC. Increased glycolytic flux, G6PD activity, and pyrimidine biosynthesis are identified in glucomet-PDAC with high GLUT1 and low ALDOB expression, and these phenotypes could be reversed by inhibiting GLUT1 expression or by increasing ALDOB expression. Pharmacological inhibition of GLUT1 or G6PD enhances the chemotherapy response of glucomet-PDAC. Our findings uncover potential metabolic heterogeneity related to differences in chemotherapy sensitivity in PDAC and develop a promising pharmacological strategy for patients with chemotherapy-resistant glucomet-PDAC through the combination of chemotherapy and GLUT1/ALDOB/G6PD axis inhibitors.

## Highlights

• Deep metabolism landscape of pancreatic ductal adenocarcinoma organoids
• Identify metabolic subtypes with prognostic significance and therapeutic implications
• Chemotherapy-resistant glucomet-PDAC depends on GLUT1/ALDOB/G6PD axis
• GLUT1/ALDOB/G6PD axis is potential target for chemotherapy-resistant glucomet-PDAC

## Graphical abstract

**Figure:**
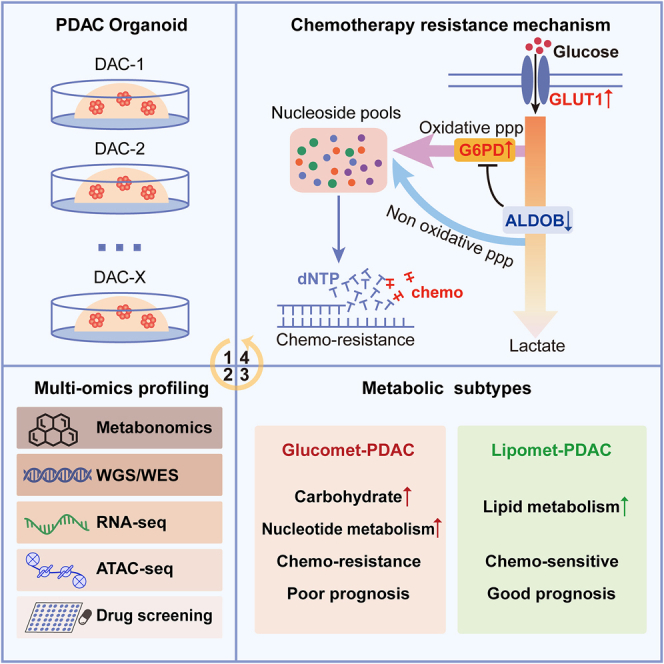

## Teaser

Li et al. conduct an integrated genetics-metabolomics analysis, classify PDAC organoids into glucomet-PDAC and lipomet-PDAC, and develop a promising pharmacological strategy for patients with chemotherapy-resistant glucomet-PDAC through the combination of chemotherapy and GLUT1/ALDOB/G6PD axis inhibitors.

## Introduction

Pancreatic ductal adenocarcinoma (PDAC) is one of the most lethal malignancies and has a 5-year survival rate of 11%.^1^ Chemotherapy could significantly prolong the survival of patients with PDAC,^2^ but the chemotherapy response rate of patients with PDAC remains low due to complex and unclear drug-resistance mechanisms.^3^^,^^4^ Although many studies have focused on the classification of PDAC based on genomic and transcriptomic signatures,^5^^,^^6^^,^^7^^,^^8^^,^^9^^,^^10^ the currently defined signatures of PDAC do not indicate chemotherapy sensitivity or guide treatment decisions. Metabolic reprogramming is recognized as an emerging mechanism of therapy resistance and presents opportunities for cancer treatment.^2^^,^^11^^,^^12^^,^^13^^,^^14^^,^^15^ However, few studies have examined the metabolic dysregulation and heterogeneity of PDAC because of the presence of abundant stromal cells, making capturing precise tumor-specific metabolite information difficult. Thus, systematically characterizing the metabolic and genomic profiles of PDAC may uncover the underlying molecular details of chemosensitivity and facilitate the development of targeted therapies to prevent or reverse chemotherapy resistance.

Since altered metabolism is one of the hallmarks of cancer, there is growing interest in the relationship between metabolism (particularly glucose metabolism) and PDAC initiation, progression, and therapy resistance.^16^^,^^17^ Previous studies have been conducted in PDAC cell lines with different metabolite levels in glycolysis, lipogenesis, and redox pathways, which exhibit distinct sensitivity to a variety of metabolic inhibitors.^18^ Furthermore, intratumoral metabolism heterogeneity within individual PDAC tumors has been identified in contributing to therapy resistance with OXPHOS inhibitors.^19^ In mouse models, KRAS mutations and hypoxia are known inducers of the glycolytic pathway in PDAC.^20^^,^^21^^,^^22^ Disruption of distal cholesterol biosynthesis by conditional inactivation of Nsdhl or by treatment with statins switches the classical phenotype to a basal phenotype in mouse models.^23^ On the basis of the median normalized expression of glycolytic and cholesterogenic genes, four metabolic expression subtypes were identified, and glycolytic tumors were associated with the shortest median survival.^24^ These studies highlight the need to characterize the metabolic signatures and identify essential pathways for PDAC cell survival and chemotherapy resistance, which may provide a therapeutic window.

Patient-derived cancer organoids have emerged as a research model and have proven superior to traditional cell lines in recapitulating the features of primary tumors.^25^^,^^26^^,^^27^^,^^28^^,^^29^^,^^30^ We have established a large PDAC organoid biobank and characterized these organoids by multiomics integration analysis.^31^ Here, we characterized the metabolic profiles of PDAC organoids and identified two metabolic subtypes, termed glucomet-PDAC (high glucose metabolism) and lipomet-PDAC (high lipid metabolism). We found that the GLUT1/aldolase B (ALDOB)/glucose-6-phosphate dehydrogenase (G6PD) axis regulates glucose metabolic reprogramming and confers chemotherapy resistance in glucomet-PDAC. Moreover, we presented a potential pharmacological strategy that involves targeting the GLUT1/ALDOB/G6PD axis to enhance the therapeutic sensitivity of glucomet-PDAC.

## Results

### Metabolite profiling stratifies PDAC into lipomet-PDAC and glucomet-PDAC

**Figure 1:**
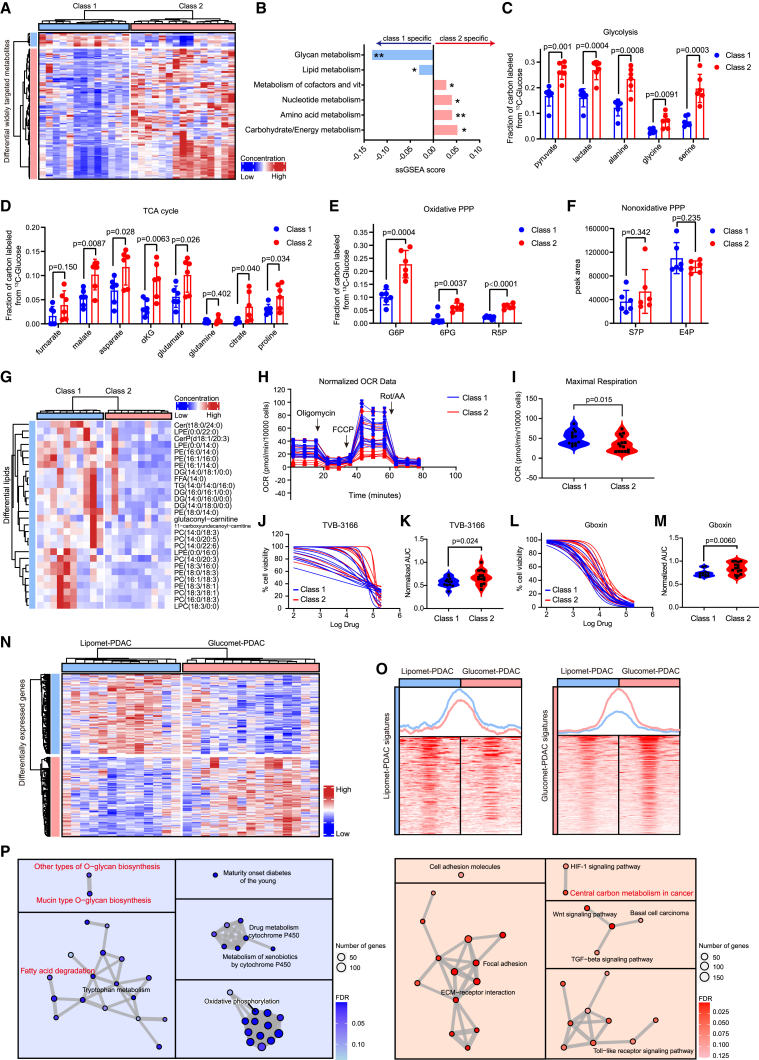
Metabolite profiling stratifies PDAC into two subtypes (A) PDAC organoid subgrouping based on widely targeted metabolite abundance. Differential metabolites were identified by the Wilcoxon rank-sum test (p < 0.05, fold change > 1.2). Samples and metabolites are displayed as columns and rows, respectively, and the color of each organoid shows the relative abundance of the metabolites. (B) Relative enrichment of the six metabolic ontology classes in class 1 and class 2 organoids, presented as the difference (class 2 versus class 1) in the ssGSEA score. Positive scores represent terms enriched in metabolites with high intensities in class 2. (C) Fractions of labeled metabolites in glycolysis from [U-^13^C_6_] glucose in representative organoids of class 1 (n = 6) and class 2 (n = 6). (D) Fractions of labeled metabolites in the TCA cycle from [U-^13^C_6_] glucose in representative organoids of class 1 (n = 6) and class 2 (n = 6). (E and F) Fractions of labeled metabolites in oxidative PPP (E) and nonoxidative PPP (F) from [U-^13^C_6_] glucose in representative organoids of class 1 (n = 6) and class 2 (n = 6). (G) Heatmap of widely targeted lipidomics assay showing the abundance of differentially lipid metabolites in class 1 (n = 6) and class 2 (n = 6). (H and I) Class 1 (n = 13) and class 2 (n = 15) organoids were exposed to oligomycin, FCCP, and rotenone/antimycin A to measure the oxygen consumption rate (OCR) by XF Cell Mito Stress Test. (J and K) Dose-response curves and normalized area under the curve (AUC) distribution for TVB-3166 (fatty acid synthase inhibitor) on class 1 and class 2. (L and M) Dose-response curves and normalized AUC distribution for Gboxin (inhibitor of oxidative phosphorylation) on class 1 (n = 13) and class 2 (n = 15). (N) Heatmap of RNA sequencing (RNA-seq) showing the expression of differentially expressed genes (DEGs) in glucomet-PDAC (n = 15) and lipomet-PDAC (n = 13) (p was calculated with limma, p < 0.05 and fold change > 1.5). Representative genes associated with lipid or glucose metabolism are shown in the box on the right. (O) Profile plot and heatmap of assay for transposase-accessible chromatin with sequencing (ATAC-seq) results showing the distribution of differential peaks around the transcriptional start site (TSS) of signature genes in lipomet-PDAC (n = 13) and glucomet-PDAC (n = 15). (P) KEGG pathways enriched in the lipomet subtype (left) and glucomet subtype (right) identified by GSEA. The significance of the difference was determined by Student’s t test (∗p < 0.05; ∗∗p < 0.01) (B). Data are presented as the mean values ± SEMs, and statistical significance was computed by unpaired Student’s t test (C–F). Statistical significance was computed by unpaired Student’s t test (I, K, and M). αKG, α-ketoglutarate; G6P, glucose-6-phosphate; 6PG, 6-phosphate gluconate; F6P, fructose-6-phosphate; R5P, ribose 5-phosphate; S7P, sedoheptulose-7-phosphate; E4P, erythrosine 4-phosphate.

To characterize the metabolic profiles of PDAC, we examined metabolites via a widely targeted metabolomics assay in 28 patient-derived PDAC organoids^31^ (Table S1). According to the consensus matrix, we identified two optimal metabolic subtypes according to targeted metabolomics (Figures 1A and S1A; Table S2), and this result was further confirmed in silhouette analysis (Figure S1B).

We calculated the enrichment score of previously established metabolic ontologies in the two metabolic subtypes by single-sample gene set enrichment analysis (ssGSEA). Class 2 organoids were characterized by marked enrichment of carbohydrate metabolism, energy metabolism, and nucleotide metabolism, indicating increased glucose metabolism (Figure 1B). We then used [U-^13^C_6_] glucose to track metabolic flux in the two metabolic subtypes and found that metabolic flux in glycolysis and the tricarboxylic acid (TCA) cycle was significantly increased in class 2 (Figures 1C and 1D). Moreover, we noted that the oxidative pentose phosphate pathway (PPP) metabolites G6P, 6PG, and R5P, but not the nonoxidative PPP metabolites S7P and E4P, were highly enriched in class 2 organoids, suggesting glucose metabolic reprogramming in class 2 (Figures 1E and 1F). Thus, we termed class 2 organoids as glucomet-PDAC. Class 1 was characterized by relatively enriched lipid metabolism (Figure 1B). We validated the lipid metabolic dependency of class 1 in three ways. Firstly, we compared the lipid metabolites of two metabolic subtypes by widely targeted lipidomics assay. We found that all significant differential lipids were enriched in class 1 (Figure 1G; Table S2). Secondly, class 1 showed higher oxygen consumption rates than glucomet-PDAC (Figures 1H and 1I). Thirdly, class 1 was more sensitive to the fatty acid synthase inhibitor and oxidative phosphorylation inhibitor (Figures 1J–1M). Therefore, these class 1 organoids were termed as lipomet-PDAC. These metabolic profiles classified PDAC into lipomet-PDAC and glucomet-PDAC with elevated lipid and glucose metabolism, respectively.

Abnormal accumulation of metabolites commonly results from the reprogramming of metabolic pathways.^32^ To identify the molecular mechanism of PDAC metabolic reprogramming, we systematically analyzed the genomic and transcriptomic profiles. Surprisingly, glucomet-PDAC and lipomet-PDAC shared similar gene mutation profiles (Figures S1C–S1E). We next generated signatures of lipomet-PDAC and glucomet-PDAC based on RNA expression data (Figure 1N; Table S3) and identified the increased chromatin accessibility of signature genes in corresponding subtypes (Figure 1O). Consistent with the differences in metabolite levels, the expression of corresponding lipid metabolism- and glycan biosynthesis-associated genes was increased in lipomet-PDAC, and the expression of genes associated with glucose metabolism (the hypoxia inducible factor-1 [HIF-1] signaling pathway and central carbon metabolism in cancer) were increased in glucomet-PDAC (Figure 1P).

### Glucomet-PDAC is associated with worse prognosis and chemoresistance

**Figure 2:**
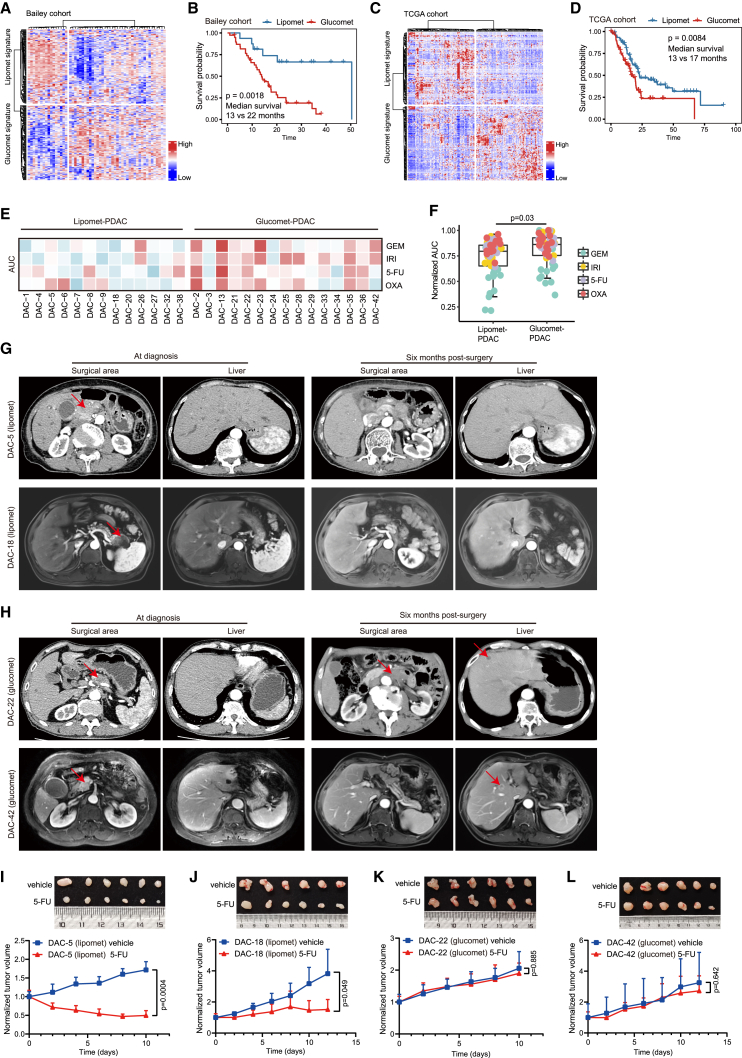
Glucomet-PDAC is associated with worse prognosis and chemoresistance (A) Heatmap of tumors in the Bailey PDAC cohort (n = 55, only squamous and pancreatic progenitor samples were included) split by glucomet and lipomet signature genes. (B) Kaplan-Meier survival curves of the Bailey PDAC cohort showing differential prognosis among patients with different subtypes. (C) Heatmap of tumors in the TCGA PDAC cohort (n = 156, only ductal pancreatic cancer samples were included) split by glucomet and lipomet signature genes. (D) Kaplan-Meier survival curves of the TCGA PDAC cohort showing differential prognosis among patients with different subtypes. (E) Normalized AUC distribution for GEM, 5-FU, OXA, and IRI on glucomet-PDAC (n = 15) and lipomet-PDAC (n = 13). The *Z* scores of the obtained normalized AUC values are depicted in the heatmap. High values (indicating resistance) are depicted in red, and low values (indicating sensitivity) are depicted in blue. (F) Comparison of AUCs of four agents among the two metabolic subtypes. The boxplot shows the median (central line) and the 25%–75% interquartile range (box limits). (G and H) Representative radiation examination of both the surgical area and liver in lipomet-PDAC (DAC-5 and DAC-18) and glucomet-PDAC (DAC-42 and DAC-22) at the time of diagnosis and 6 months postsurgery. The arrow marks the tumor position. (I–L) 5-FU responsiveness test in the indicated ODX models (n = 6 per group). Tumor volumes measured by calipers at the indicated time points in tumor-implanted mice subjected to treatments with control or 5-FU (25 mg/kg, every 2 days). Statistical significance was computed by log rank test (B and D). Significance was computed by a one-sided paired t test (F). Data are presented as the mean values ± SEMs, and statistical significance was computed by unpaired Student’s t test (I–L). GEM, gemcitabine; 5-FU, 5-fluorouracil; IRI, irinotecan; OXA, oxaliplatin.

We next investigated whether the metabolic subtypes were associated with clinical outcomes. In the absence of appropriate longitudinal data, the Bailey PDAC cohort (n = 55 patients) and the TCGA PDAC cohort (n = 156 patients) were classified into two subtypes based on the expression of metabolic signature genes.^8^^,^^33^ Patients with glucomet-PDAC showed significantly worse overall survival than patients with lipomet-PDAC in the Bailey PDAC cohort and the TCGA PDAC cohort (Figures 2A–2D). As expected, cases identified as the basal subtype based on RNA data had a markedly worse prognosis than those identified as the classical subtype (Figures S1F and S1G). Strikingly, metabolic subtypes performed better than RNA subtypes in stratifying patients based on prognosis.

We further assessed the chemotherapy sensitivity of glucomet-PDAC and lipomet-PDAC using gemcitabine (GEM), 5-fluorouracil (5-FU), irinotecan (IRI), and oxaliplatin (OXA) (four chemotherapeutic drugs that work by inhibiting DNA synthesis), which are commonly used to treat patients with PDAC (Figures S1H–S1K; Table S4). Therapeutic profiling revealed marked interpatient variability in the response to a single chemotherapy agent, but glucomet-PDAC organoids were generally more resistant to chemotherapeutic agents than lipomet-PDAC organoids (Figures 2E and 2F). To determine whether PDAC organoids can precisely reflect the chemosensitivity of patients, we obtained clinical follow-up data from four patients with PDAC with 5-FU as adjuvant therapy after surgery. Two patients with glucomet-PDAC (corresponding to two organoid lines: DAC-42 and DAC-22) relapsed 6 months after surgery, while the other two patients with lipomet-PDAC (corresponding to two organoid lines: DAC-5 and DAC-18) were disease free 6 months after surgery (Figures 2G and 2H). Glucomet-PDAC and lipomet-PDAC organoid-derived xenografts (ODXs) were used to evaluate chemosensitivity *in vivo*. 5-FU treatment significantly inhibited the growth of lipomet-PDAC ODXs but had no significant effects on 5-FU-resistant, glucomet-PDAC-derived xenografts (Figures 2I–2L). These findings suggest that glucomet-PDAC is chemoresistant and that it is associated with worse prognosis.

### The GLUT1/ALDOB/G6PD axis reprograms glucose metabolism in PDAC

**Figure 3:**
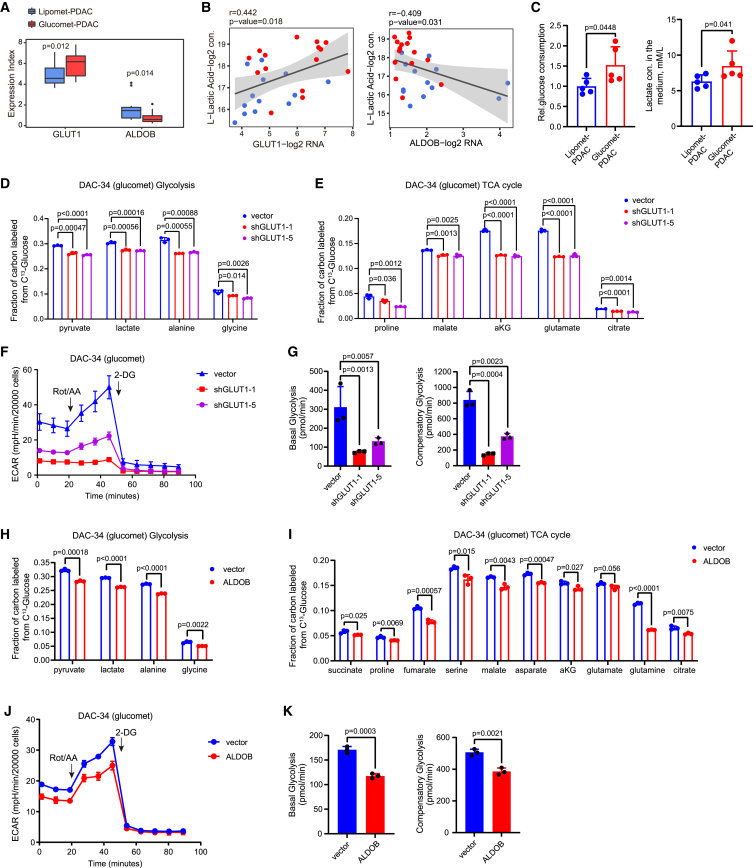
The GLUT1 and ALDOB drives glucose metabolic reprogramming in glucomet-PDAC (A) Boxplot of *ALDOB* and *GLUT1* expression levels stratified by metabolic subgroup (p was calculated with limma). (B) Scatterplot showing the correlation of lactate intensities and *GLUT1* or *ALDOB* gene expression (Pearson correlation analysis). Each dot represents an individual sample. Color represents the metabolic subgroup (red dot represents glucomet-PDAC, and blue dot represents lipomet-PDAC). (C) Extracellular glucose consumption and lactate secretion were evaluated in representative organoids of glucomet-PDAC (n = 5) and lipomet-PDAC (n = 5). (D and E) Fractions of labeled metabolites in glycolysis (D) and the TCA cycle (E) from [U-^13^C_6_] glucose in control and *GLUT1* knockdown organoids (n = 3 per group). (F and G) Control and *GLUT1* knockdown organoids were exposed to rotenone/antimycin A and 2-DG to measure the ECAR at the basal level and compensatory level by the Seahorse XF Glycolytic Rate Assay (n = 3 per group). (H and I) Fractions of labeled metabolites in glycolysis (H) and the TCA cycle (I) from [U-^13^C_6_] glucose in control and *ALDOB*-overexpressing organoids (n = 3 per group). (J and K) Control and *ALDOB*-overexpressing organoids were exposed to rotenone/antimycin A and 2-DG to measure the ECAR at the basal level and compensatory level by the Seahorse XF Glycolytic Rate Assay (n = 3 per group). (C–E, G–I, and K) Data are presented as mean values ± SEMs. Statistical significance was computed by unpaired Student’s t test.

Transcriptional differences within metabolic pathways could indicate the glucose reprogramming of PDAC. We noted that the expression of metabolic genes in glycolysis, the glucose transporter *GLUT1* (also known as *SLC2A1*) and the aldehyde dehydrogenase *ALDH1A3*, was upregulated in glucomet-PDAC compared with lipomet-PDAC. Intriguingly, the expression of the metabolic enzyme *ALDOB* in glycolysis was decreased in glucomet-PDAC compared with lipomet-PDAC (Figure 3A). GLUT1, which is a viable drug target and a predictor of worse prognosis,^34^^,^^35^ is the main classical transporter for glucose uptake in tumors.^36^^,^^37^^,^^38^^,^^39^ Cancer cells activate aerobic glycolysis and convert the majority of glucose into lactate.^40^ As expected, lactate levels were positively correlated with *GLUT1* expression in PDAC organoids (Figure 3B). Furthermore, increased glucose uptake and lactate secretion were identified in glucomet-PDAC compared with lipomet-PDAC (Figure 3C). Although our PDAC organoid lines have the similar growth rates between glucomet-PDAC and lipomet-PDAC both *in vitro* and upon transplantation *in vivo* (Figures S2A and S2B), the promoting effect of glucose on organoid growth was significantly greater for glucomet-PDAC than for lipomet-PDAC (Figures S2C and S2D). We used [U-^13^C_6_] glucose to track metabolic flux in PDAC organoids and found that *GLUT1* knockdown significantly decreased the incorporation of [U-^13^C_6_] glucose into glycolysis and the TCA cycle (Figures 3D, 3E, and S2E–S2H). Consistently, in comparison with the control organoids, *GLUT1* knockdown organoids demonstrated a decreased extracellular acidification rate (ECAR) (Figures 3F, 3G, S2I, and S2J).

We applied metabolon-based energy metabolism analysis methods to further characterize differences in glucose metabolism between lipomet-PDAC and glucomet-PDAC. Four metabolites in the glycolytic pathway presented significantly higher intensities in glucomet-PDAC than in lipomet-PDAC. Increased levels of ALDOB upstream metabolites (G6P, F6P, and F1,6BP) and decreased *ALDOB* expression favored a model of glucose metabolic reprogramming in glucomet-PDAC with loss of *ALDOB* (Figures 3A and S2K). Moreover, *ALDOB* expression was negatively correlated with lactate levels (Figure 3B). We found that *ALDOB* overexpression significantly decreased the incorporation of [U-^13^C_6_] glucose into glycolysis and the TCA cycle (Figures 3H, 3I, S2L, and S2M). Consistently, *ALDOB* overexpression led to a significant decrease in basal glycolysis and compensatory glycolysis in glucomet-PDAC organoids (Figures 3J, 3K, S2N, and S2O).

**Figure 4:**
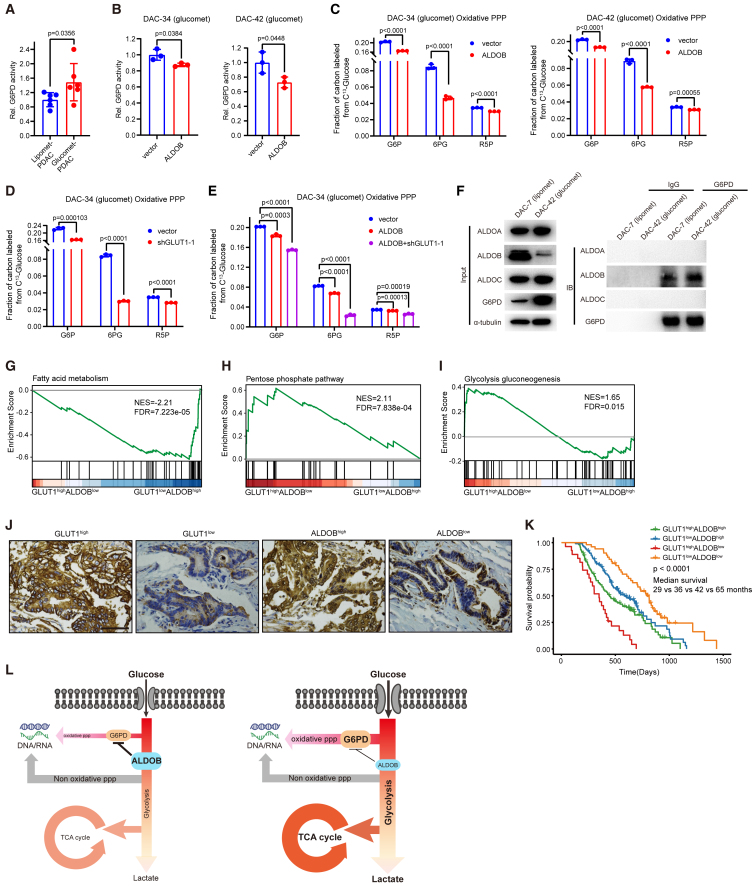
The GLUT1/ALDOB/G6PD axis drives glucose metabolic reprogramming in glucomet-PDAC (A) Relative G6PD enzyme activity in representative organoids of glucomet-PDAC (n = 6) and lipomet-PDAC (n = 6). (B) Relative G6PD enzyme activity in vector- and *ALDOB*-overexpressing organoids (n = 3). (C) Relative abundance of oxidative PPP metabolites in control and *ALDOB*-overexpressing organoids (n = 3). (D and E) Relative abundance of oxidative PPP metabolites in control, *GLUT1* knockdown, *ALDOB* overexpression, *GLUT1* knockdown, and *ALDOB* overexpression organoids (n = 3). (F) Endogenous ALDOB and G6PD interactions in PDAC organoids detected by immunoprecipitation (IP) experiments. (G–I) TCGA patients with PDAC (n = 156) were divided into *GLUT1*^high^/*ALDOB*^low^, *GLUT1*^low^/*ALDOB*^high^, and others based on *GLUT1* and *ALDOB* expression levels. Samples with expression of *GLUT1* in the top 40% and expression of *ALDOB* in the last 40% were named *GLUT1*^high^/*ALDOB*^low^, while samples with expression of *ALDOB* in the top 40% and expression of *GLUT1* in the last 40% were named *GLUT1*^low^/*ALDOB*^high^. GSEA enrichment plot for *GLUT1*^high^/*ALDOB*^low^ group versus *GLUT1*^low^/*ALDOB*^high^ group of fatty acid metabolism (G), pentose phosphate pathway (H), and glycolysis/gluconeogenesis (I) signature genes. (J) Representative images of immunohistochemical staining show high or low GLUT1 staining and high or low ALDOB staining in PDAC TMA (n = 285). Scale bar, 50 μm. Patients were divided into four groups based on ALDOB and GLUT1 expression levels: ALDOB^high^ (++), ALDOB expression >50%; ALDOB^low^ (+), ALDOB expression <50%; GLUT1^high^ (++), GLUT1 expression >50%; and GLUT1^low^ (+), GLUT1 expression <50%. (K) Kaplan-Meier survival curves based on the expression of GLUT1 and ALDOB in 285 patients with PDAC. (L) Summary scheme highlighting the roles of the GLUT1/ALDOB/G6PD axis in glucose reprogramming. Data are presented as the mean values ± SEMs, and statistical significance was computed by unpaired Student’s t test (A–E). Statistical significance was computed by log-rank test (K).

Previously, we found that Aldob regulated glucose metabolism and suppressed hepatocellular carcinogenesis (HCC) by inhibiting G6PD activity through direct interaction.^41^ Elevated oxidative PPP metabolite levels and G6PD enzyme activity were found in glucomet-PDAC (Figures 1E and 4A). *ALDOB* overexpression significantly inhibited G6PD enzyme activity and decreased oxidative PPP metabolite levels in glucomet-PDAC organoids (Figures 4B and 4C). Furthermore, *GLUT1* knockdown further suppressed the oxidative PPP pathway in glucomet-PDAC organoids under *ALDOB* overexpression conditions (Figures 4D and 4E). To determine whether ALDOB inhibits G6PD activity by direct interaction in PDAC organoids, the interaction between ALDOB and G6PD was investigated by immunoprecipitation analysis. We found that G6PD pulled down ALDOB, but not ALDOA or ALDOC, in two representative organoids (Figure 4F). These results indicate that low ALDOB expression is critical for the high oxidative PPP levels of glucomet-PDAC.

We expanded the analysis to 156 patients by a larger cohort from TCGA to validate our results. GSEA was performed using TCGA data from patients with PDAC based on *GLUT1* and *ALDOB* transcriptional levels. Fatty acid metabolism pathway genes were significantly enriched in the low *GLUT1* with high *ALDOB* (*GLUT1*^low^/*ALDOB*^high^) expression group (Figure 4G). PPP metabolism and glycolysis pathway genes were enriched in the high *GLUT1* with low *ALDOB* (*GLUT1*^high^/*ALDOB*^low^) expression group (Figures 4H and 4I).

Then, we performed tissue microarray (TMA) analyses on patient samples (n = 283) with complete clinical data to assess the association between GLUT1/ALDOB protein expression and clinical outcomes. GLUT1 or ALDOB protein expression scores were classified as low or high (Figure 4J; Table S5). In the Kaplan-Meier analysis, patients with GLUT1^high^/ALDOB^low^ protein expression had the worst overall survival, whereas patients with GLUT1^low^/ALDOB^low^ protein expression had the best overall survival (Figure 4K). These results indicated that patients with glucomet-PDAC (GLUT1^high^/ALDOB^low^) have a worse prognosis.

To confirm the potential of GLUT1 and ALDOB as predictive biomarkers of therapeutic response at the RNA and protein levels, we analyzed the published PDAC cohort,^42^ which has both RNA and protein expression results. We classified this PDAC cohort into two subtypes based on the RNA and protein expression of metabolic signature genes, respectively. As expected, a notable gene expression trend of GLUT1^high^/ALDOB^low^ was identified in glucomet-PDAC based on RNA and protein expression levels (Figures S3A–S3D). Patients with glucomet-PDAC showed worse overall survival than patients with lipomet-PDAC in the cohort (Figures S3E and S3F). GLUT1^high^/ALDOB^low^ patients also showed significantly worse overall survival in the cohort (Figures S3G and S3H). These results confirmed the value of the GLUT1/ALDOB axis as a biomarker of drug response and prognosis, and both protein and RNA could be applied to predict drug response.

These results suggest an essential role for the GLUT1/ALDOB/G6PD axis in regulating metabolic reprogramming, which enhances glucose entry into glycolysis, the TCA cycle, and the oxidative PPP in glucomet-PDAC (Figure 4L).

### GLUT1/ALDOB/G6PD axis-mediated chemoresistance by increasing pyrimidine nucleosides

**Figure 5:**
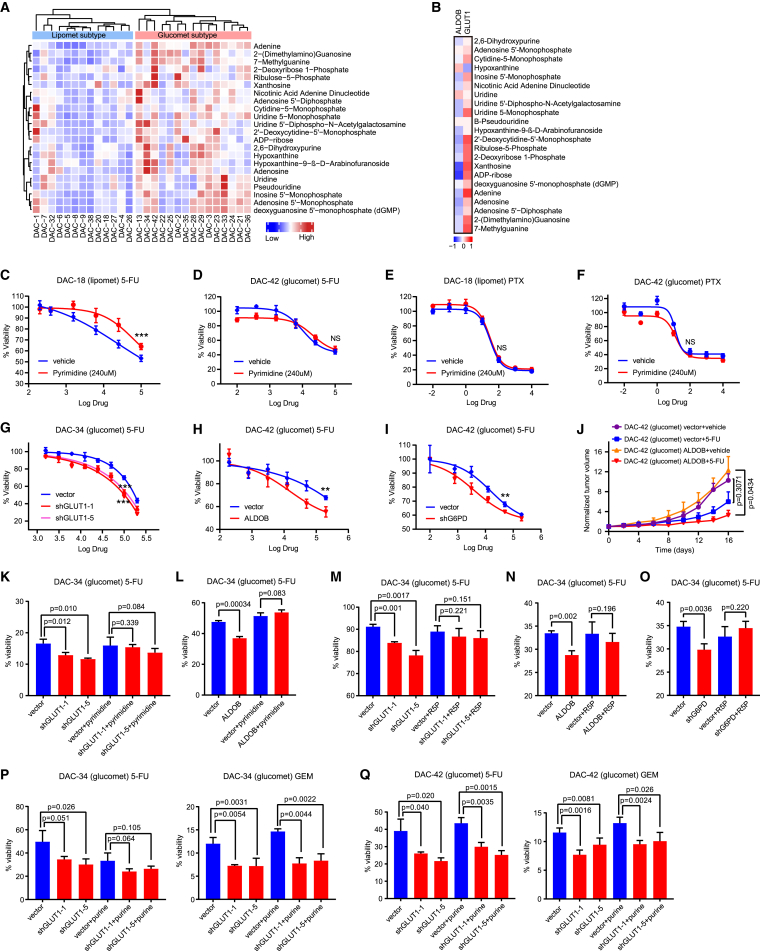
The GLUT1/ALDOB/G6PD axis contributes to drug resistance (A) Heatmap showing the relative abundance of nucleoside and nucleoside derivatives in glucomet-PDAC (n = 15) and lipomet-PDAC (n = 13). (B) Heatmap showing Pearson correlation coefficients between the RNA expression levels of *ALDOB*/*GLUT1* and nucleoside derivatives. (C and D) DAC-18 (lipomet) and DAC-42 (glucomet) organoids were treated with 5-FU alone or in combination with pyrimidine nucleosides (uridine, cytidine, and thymidine, 240 μM) for 5 days, and cell viability was determined by CellTiter-Glo assays. (E and F) DAC-18 (lipomet) and DAC-42 (glucomet) organoids were treated with PTX alone or in combination with pyrimidine nucleosides (240 μM) for 5 days, and cell viability was determined by CellTiter-Glo assays. (G) Effect of *GLUT1* knockdown on 5-FU responsiveness of DAC-34 (glucomet) as determined by CellTiter-Glo assays 120 h after treatment with 5-FU. (H) Effect of *ALDOB* overexpression on the 5-FU responsiveness of DAC-42 (glucomet) as determined by CellTiter-Glo assays 120 h after treatment with 5-FU. (I) Effect of *G6PD* knockdown on the 5-FU responsiveness of DAC-42 (glucomet) as determined by CellTiter-Glo assays 120 h after treatment with 5-FU. (J) Effect of *ALDOB* overexpression on 5-FU responsiveness in the DAC-42 (glucomet) ODX model (n = 6 per group). (K) Effect of pyrimidine nucleotide (240 μM) on 5-FU sensitivity in control and *GLUT1* knockdown organoids by CellTiter-Glo assays at 120 h posttreatment. (L) Effect of pyrimidine nucleotide (240 μM) on 5-FU sensitivity in control and *ALDOB* overexpression organoids by CellTiter-Glo assays at 120 h posttreatment. (M) Effect of R5P (1 mM) on 5-FU sensitivity in control and *GLUT1* knockdown organoids by CellTiter-Glo assays at 120 h posttreatment. (N) Effect of R5P (1 mM) on 5-FU sensitivity in control and *ALDOB*-overexpressing organoids by CellTiter-Glo assays at 120 h posttreatment. (O) Effect of R5P (1 mM) on 5-FU sensitivity in control and *G6PD* knockdown organoids by CellTiter-Glo assays at 120 h posttreatment. (P and Q) Effect of purine nucleotides (guanosine and adenosine, 200 μM) on 5-FU and GEM sensitivity in control and *GLUT1* knockdown organoids by CellTiter-Glo assays at 120 h posttreatment. All dose-responsive curves were performed with 3 technical replicates. Data are presented as the mean values ± SEMs, and statistical significance was computed by unpaired Student’s t test (∗p < 0.05; ∗∗p < 0.01; ∗∗∗p < 0.001) (C–Q).

Increased flux of glycolysis and the PPP leads to an increase in nucleoside biosynthesis, including the synthesis of pyrimidine and purine nucleosides, which serve as important inducers of drug resistance.^2^^,^^12^ Widely targeted metabolomics analysis indicated that pyrimidine and purine pathway-related metabolites were enriched in glucomet-PDAC (Figure S4A). Increased levels of nucleosides and nucleoside derivatives were detected in glucomet-PDAC compared with lipomet-PDAC (Figure 5A). Furthermore, nucleoside and nucleoside derivative levels were correlated with high *GLUT1* expression and low *ALDOB* expression (Figure 5B).

To further investigate how the increased flux of the oxidative PPP regulates drug sensitivity, we assessed several key PPP metabolites, R5P, pyrimidine nucleosides, and purine nucleosides, which could be taken up by organoids from the media. Activated G6PD and increased flux of oxidative PPP provide R5P for nucleotide biosynthesis. R5P is essential for DNA replication and DNA damage repair. R5P significantly enhanced 5-FU resistance in lipomet-PDAC organoids but had no effects on the 5-FU treatment response in glucomet-PDAC organoids (Figures S4B and S4C). Interestingly, exogenous addition of pyrimidine nucleosides, but not purine nucleosides, induced therapeutic resistance to GEM and 5-FU in lipomet-PDAC organoids but had no effects on glucomet-PDAC organoids (Figures 5C, 5D, and S4D–S4G). However, increased levels of pyrimidine nucleosides did not affect the sensitivity to nab-paclitaxel (PTX; acting on microtubules) in lipomet-PDAC and glucomet-PDAC (Figures 5E and 5F). These results showed that the increased flux of glucose into the pyrimidine nucleoside biosynthesis pathway led to chemotherapy resistance in glucomet-PDAC.

*GLUT1* knockdown, *ALDOB* overexpression, or *G6PD* knockdown distinctly increased the sensitivity to GEM, 5-FU, IRI, and OXA in glucomet-PDAC organoids (Figures 5G–5I and S5A–S5E). Moreover, *ALDOB* overexpression significantly increased the sensitivity to 5-FU in glucomet-PDAC *in vivo* (Figures 5J and S5F). However, expression changes in *GLUT1* or *ALDOB* did not affect the sensitivity to PTX in glucomet-PDAC organoids (Figures S5G–S5J).

The addition of exogenous pyrimidine nucleosides and R5P effectively rescued the increase in drug sensitivity induced by *GLUT1* knockdown, *ALDOB* overexpression, or *G6PD* knockdown in glucomet-PDAC cells (Figures 5K–5O and S5K–S5M). However, the addition of exogenous purine nucleosides had no effects on chemotherapy sensitivity in glucomet-PDAC organoids under *GLUT1* knockdown conditions (Figures 5P and 5Q). Furthermore, *ALDOB* knockdown distinctly induced chemotherapy resistance to 5-FU and GEM in lipomet-PDAC organoids (Figures S5N–S5P). The addition of exogenous pyrimidine nucleosides effectively decreased the chemosensitivity difference in lipomet-PDAC organoids between the *ALDOB* knockdown and control groups (Figures S5O and S5P). These results revealed that the GLUT1/ALDOB/G6PD axis mediated chemoresistance by modulating glucose metabolism and the levels of pyrimidine nucleosides in pancreatic cancer cells.

### Pharmacological inhibition of the GLUT1/ALDOB/G6PD axis enhances chemotherapeutic sensitivity

**Figure 6:**
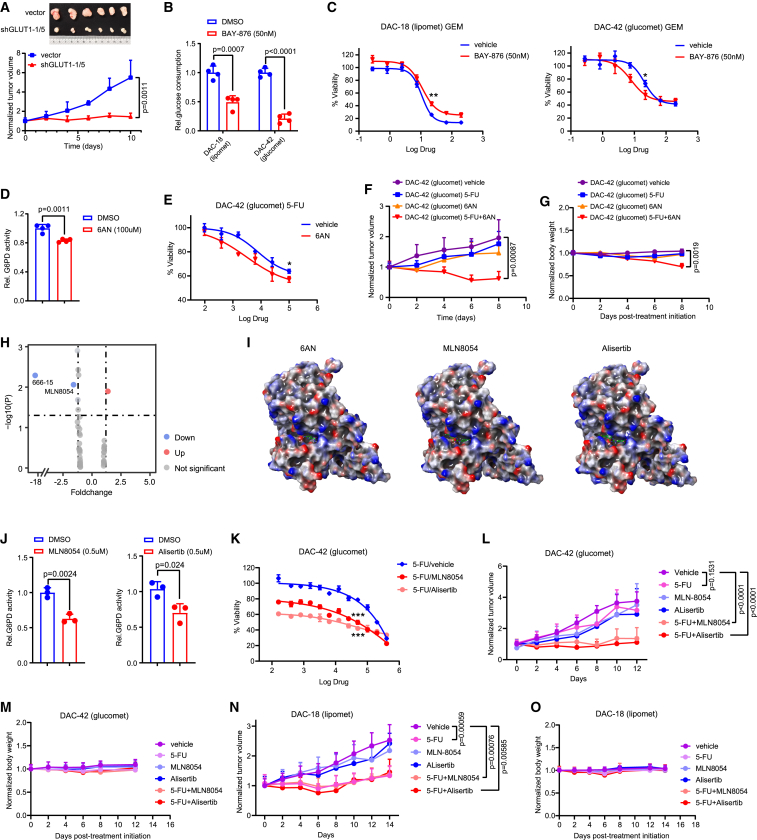
Pharmacological inhibition of GLUT1 or G6PD enhances chemotherapy sensitivity (A) *GLUT1* knockdown suppresses tumorigenesis in a DAC-42 ODX model (n = 6 per group). (B) The effect of the GLUT1 inhibitor BAY-876 (50 nM) on extracellular glucose consumption in DAC-18 (lipomet) and DAC-42 (glucomet) organoids was evaluated (n = 4). (C) Effect of BAY-876 (50 nM) on the GEM responsiveness of DAC-42 (glucomet) and DAC-18 (lipomet) as determined by CellTiter-Glo assays 120 h after treatment with GEM. (D) The effect of 6AN on G6PD activity in DAC-42 (glucomet) organoids was evaluated (n = 4). (E) Effect of 6AN treatment on the 5-FU responsiveness of DAC-42 (glucomet) organoids as determined by CellTiter-Glo assays 120 h after treatment with 5-FU. (F) Effect of 6AN on 5-FU responsiveness in the ODX model. Tumor volumes measured by calipers at the indicated time points in tumor-implanted mice subjected to treatments with vehicle, 5-FU alone (25 mg/kg, every 2 days), 6AN alone (5 mg/kg, every 2 days), or 5-FU with 6AN. (G) Normalized body weights of SCID mice with the indicated treatments. (H) Volcano plot showing the effect of 40 compounds on the 5-FU responsiveness of DAC-34 (glucomet) as determined by CellTiter-Glo assays 120 h after treatment with compounds (5 μM), 5-FU (100 μM), or both. (I) Prediction of the potential interaction sites in the 3D structure of the G6PD protein with 6AN, MLN8054, and alisertib. (J) Effect of MLN8054 and alisertib on G6PD activity in DAC-42 (glucomet) organoids. (K) Effect of MLN8054 or alisertib treatment on the 5-FU responsiveness of DAC-42 (glucomet) as determined by CellTiter-Glo assays 120 h after treatment with the indicated drugs. (L) Tumor volume of DAC-42 ODXs (n = 6 per group) in SCID mice following treatment with the indicated drugs. (M) Normalized body weights of SCID mice (n = 3 per group) with the indicated treatments. (N) Tumor volume of DAC-18 ODXs (n = 6 per group) in SCID mice following treatment with the indicated drugs. (O) Normalized body weights of SCID mice (n = 3 per group) with the indicated treatments. All dose-responsive curves were performed with 3 technical replicates. Data are presented as the mean values ± SEMs, and statistical significance was computed by unpaired Student’s t test (∗p < 0.05; ∗∗p < 0.01; ∗∗∗p < 0.001) (A–G and J–O).

*GLUT1* knockdown significantly inhibited glucomet-PDAC-derived xenograft growth (Figure 6A). The GLUT1 inhibitor BAY-876 decreased glucose uptake in both glucomet-PDAC organoids and lipomet-PDAC organoids (Figure 6B). BAY-876 increased the sensitivity to GEM in glucomet organoids but had no distinct effects on lipomet-PDAC organoids (Figure 6C). The addition of exogenous pyrimidine nucleosides effectively decreased the sensitivity to GEM and 5-FU in glucomet-PDAC organoids under the condition of GLUT1 inhibition by BAY-876 (Figures S6A and S6B). These results indicate that clinical-grade inhibitors of GLUT1 have the potential to enhance the chemotherapy response of PDAC.

G6PD plays an essential role in regulating the oxidative PPP pathway and nucleotide biosynthesis.^43^ We next examined the therapeutic potential of the G6PD inhibitor 6AN in promoting chemotherapy sensitivity. 6AN treatment attenuated G6PD activity but had no effect on cell viability in glucomet-PDAC and lipomet-PDAC organoids (Figures 6D and S6C). Furthermore, 6AN increased the sensitivity to 5-FU and GEM in glucomet-PDAC organoids but had no effects on lipomet-PDAC organoids (Figures 6E and S6D–S6F). 6AN and 5-FU combination significantly reduced tumor growth in chemoresistant glucomet-PDAC ODXs but seriously affected mice body weight (Figures 6F, 6G, and S6G). Considering the physiological toxicity of 6AN, we performed virtual screening of 5 compound libraries (7,098 compounds) to find candidate inhibitors of G6PD. Forty compounds were chosen as G6PD candidate inhibitors, and most of them were clinical-grade inhibitors. Among these 40 compounds, 38 were excluded based on their low inhibition rates (Figure 6H). Although the binding site of the remaining two compounds (MLN8054 and 666-15) on G6PD was the same as that of 6AN (Figures 6I and S6H), only MLN8054 significantly inhibited G6PD activity (Figures 6J and S6I). MLN8054 increased the sensitivity to 5-FU in glucomet-PDAC organoids *in vitro* (Figures 6K and S6J). To further determine the effects of MLN8054 on tumor growth *in vivo*, glucomet-PDAC DAC-42 ODXs were subjected to vehicle, 5-FU, MLN8054, or combined treatment. Without affecting body weight, combined treatment significantly suppressed tumor growth compared with vehicle or individual agent treatment (Figures 6L, 6M, and S6K). However, combined treatment did not affect the sensitivity to 5-FU in lipomet-PDAC organoids (Figures 6N, 6O, and S6L). MLN8054 and alisertib (a MLN8054 analog) have been reported as orally active small molecules that selectively inhibit Aurora A kinase and that have advanced into human clinical trials.^44^^,^^45^^,^^46^ Treatment with alisertib showed similar results to MLN8054 treatment (Figures 6J–6M). Our study discovered two clinical-grade G6PD inhibitors that significantly enhanced the chemotherapy response of PDAC.

These results indicate that targeting the GLUT1/ALDOB/G6PD axis in combination with chemotherapy has the potential to increase therapy response and survival in patients with PDAC.

## Discussion

Although chemotherapies significantly prolong survival of patients with PDAC patient, the chemotherapy response rates of PDAC remain poor.^2^^,^^47^ An effective classification strategy to divide patients into sensitive and resistance groups is urgently needed. Here, we classified patients with PDAC into glucomet-PDAC and lipomet-PDAC groups based on metabolomic profiles, identified glucomet-PDAC as a chemoresistant group, and developed a potential pharmacological strategy that involves inhibiting the GLUT1/ALDOB/G6PD axis to enhance the chemotherapy sensitivity of glucomet-PDAC.

There is increasing recognition of metabolic reprogramming as an emerging mechanism of cancer therapy resistance, but it remains poorly characterized in PDAC.^16^^,^^17^^,^^48^ Patient-derived PDAC tumor specimens usually contain an abundant stroma intermixed with normal pancreatic cells, which affects the sensitivity to characterize the PDAC cancer cells.^49^ Patient-derived cancer organoid models faithfully recapitulate the characteristics of the original tumor cells and are attractive candidates to investigate the metabolic reprogramming of PDAC.^25^^,^^26^^,^^50^ Patient-derived PDAC organoids include both basal-like and classical PDAC subtypes.^27^^,^^28^^,^^29^^,^^30^^,^^31^ Moreover, metabolic profiling of early and late recurrent PDAC using patient-derived organoids provides insight into PDAC recurrence from a metabolic perspective.^51^ Here, we successfully identified metabolic subtypes by profiling patient-derived PDAC organoids and classified them into glucomet-PDAC and lipomet-PDAC. Glucomet-PDAC organoids were resistant to chemotherapy by remodeling glucose metabolism. Importantly, the glucomet-PDAC gene expression signature GLUT1^high^/ALDOB^low^ could efficiently identify patients with the best overall survival and the worst overall survival. Our study provides an example of metabolic reprogramming as a chemotherapy resistance mechanism in PDAC and identifies markers for predicting the chemotherapy response.

Multiple molecular mechanisms of PDAC chemoresistance have been suggested,^2^^,^^52^^,^^53^^,^^54^ related to drug transport and drug metabolism. hENT1 and GATA6 expression has been associated with GEM and 5-FU sensitivity.^55^^,^^56^ However, there is no significant correlation between these two genes’ expression with GEM and 5-FU sensitivity in our cohort. Consistently, previous study has demonstrated that hENT1 expression is not reduced in some GEM-resistant PDAC cell lines.^57^ These results suggest that hENT1 expression alone may not be sufficient to predict GEM sensitivity. Increased glycolytic flux has been identified as a widely prevalent mechanism of resistance to GEM in pancreatic cancer.^12^ HIF-1α-mediated metabolic reprogramming enhanced the intrinsic levels of deoxycytidine triphosphate (dCTP).^12^ Here, we presented a mechanism of chemoresistance by remodeling glucose metabolism in glucomet-PDAC through the GLUT1/ALDOB/G6PD axis. High GLUT1-induced glucose uptake not only feeds into the glycolysis pathway but also feeds into intermediate pathways to generate biomass. Downregulation of ALDOB in glucomet-PDAC released inhibition on G6PD and oxidative PPP, resulting in increased PPP flux to meet bioenergetic and biosynthetic demands. This new metabolic rerouting strategy enables glucomet-PDAC organoids to expand intracellular pyrimidine pools that can in turn render chemotherapy ineffective by molecular competition (Figure S6M). Consistently, therapies targeting the GLUT1/ALDOB/G6PD axis are only effective when combined with chemotherapeutic agents that act on DNA synthesis.

Glucose metabolism reprogramming in cancer cells is required to fulfill anabolic demands, which provide therapeutic targets.^11^^,^^58^ Targeted inhibition of the GLUT1/ALDOB/G6PD axis in combination with chemotherapy significantly increased the therapeutic response of glucomet-PDAC. GLUT1, which has a high affinity for glucose, is overexpressed in many cancers.^36^^,^^37^^,^^38^^,^^39^ Our study and previous studies have demonstrated that the high expression level of GLUT1 correlated with chemotherapy resistance in pancreatic tumor cells.^12^ Several small molecules that inhibit GLUT1 have been shown to selectively kill cancer cells *in vitro.*^59^^,^^60^ In our study, genetic knockdown of *GLUT1* or chemical inhibition of GLUT1 activity suppressed cell viability and tumor progression both *in vitro* and *in vivo*. However, the widespread expression of *GLUT1* in different types of normal mammalian cells may preclude the clinical use of these inhibitors.^61^^,^^62^^,^^63^ The roles of G6PD and PPP have been increasingly recognized in various cancers, and G6PD upregulation is correlated with poor prognosis.^64^^,^^65^^,^^66^^,^^67^^,^^68^ G6PD inhibition significantly increased the therapeutic response to chemotherapy in glucomet-PDAC. The genetic defect of *GLUT1* or *G6PD* causes a spectrum of diseases, but the vast majority of persons with *G6PD* deficiency also may be asymptomatic.^69^^,^^70^ Therefore, clinical-grade G6PD inhibitors may have the potential to improve the PDAC treatment response.

Overall, our study provides a new PDAC classification strategy based on metabolic profiles and reports that the GLUT1/ALDOB/G6PD axis induces chemoresistance by remodeling glucose metabolism in glucomet-PDAC. These results provide a strong rationale for future drug development and clinical trials designed to target the GLUT1/ALDOB/G6PD axis to overcome metabolic reprogramming-driven chemotherapy resistance.

### Limitations of the study

PDAC organoids offer a pure cancer cell population for investigating the molecular mechanism of chemotherapy resistance; however, chemotherapy resistance is multifactorial, which also includes the cells of tumor microenvironment. Therefore, a more comprehensive chemotherapy resistance mechanism from cancer cell and microenvironment interaction may be essential in the future. Our study identified the core glucose metabolic alterations that mediate chemotherapy resistance in intrinsically resistant PDAC organoids. Whether these metabolic alterations also contribute to acquired chemotherapy resistance still needs to be identified. In addition, we report the potential role of MLN8054 and alisertib in inhibiting G6PD activity and increasing sensitivity of PDAC organoids. However, whether these two inhibitors really benefit patients with PDAC clinically needs more clinic studies in the future.

## STAR★Methods

### Key resources table

**Table:** 

REAGENT or RESOURCE	SOURCE	IDENTIFIER
**Antibodies**		

GLUT1	abcam	Cat#ab115730; RRID:AB_10903230
ALDOA	proteintech	Cat#11217-1-AP; RRID:AB_2224626
ALDOB	proteintech	Cat#18065-1-AP; RRID:AB_2273968
ALDOC	proteintech	Cat#14884-1-AP; RRID:AB_2226691
G6PD	abcam	Cat#ab993; RRID:AB_296714
α-tubulin	proteintech	Cat#11224-1-AP; RRID:AB_2210206
actin	abmart	Cat#M20011; RRID:AB_2936240

**Biological samples**		

Human tissue samples for sequencing analyses	This study	N/A
Human blood samples for sequence analyses	This study	N/A

**Chemicals, peptides, and recombinant proteins**		

Advanced DMEM/F12	Gibco	Cat#12634010
HEPES	Gibco	Cat#15630080
GlutMAX	Gibco	Cat#35050061
Penicillin-Streptomycin	Gibco	Cat#15140122
Primocin	InvivoGen	Cat#ant-pm-2
B27 supplement	Gibco	Cat#17504044
Wnt3A conditioned medium	Gao lab	N/A
R-spondin conditioned medium	Gao lab	N/A
Noggin conditioned medium	Gao lab	N/A
Nicotinamide	SIGMA	Cat#N0636-100G
EGF	Invitrogen	Cat#PHG0313
FGF10	PEPRO TECH	Cat#K2717
N-acetyl-L-cysteine	SIGMA	Cat#A9165-100G
A83-01	TOCRIS	Cat#2939
Y-27632	Selleck chemicals	Cat#S1049
Forskolin	Selleck chemicals	Cat#S2449
TrypLE™ Express	Gibco	Cat#12605028
Collagenase Type II	Gibco	Cat#17101015
Matrigel Matrix (For organoid culture)	CORNING	Cat#356231
Matrigel Matrix (For xenograft)	CORNING	Cat#354234
Gemcitabine	TargetMol	Cat#T0251
Paclitaxel	Selleck chemicals	Cat#S1150
5-fluorouracil	SIGMA	Cat#F6627
Oxaliplatin	Selleck chemicals	Cat#S1124
Irinotecan	Selleck chemicals	Cat#S1198
BAY-876	Selleck chemicals	Cat#S8452
6AN	Selleck chemicals	Cat#S9783
MLN8054	TargetMol	Cat#T6315
Alisertib	Selleck chemicals	Cat#S1133
Uridine	Sigma	Cat#U3003
Cytidine	Sigma	Cat#C4654
Thymidine	Sigma	Cat#T1895
Guanosine	Sigma	Cat#G6264
adenosine	Sigma	Cat#A4036
R5P	MCE	Cat#207671-46-3
CellTiter-Glo Luminescent	Promega	Cat#G7573
DMSO	Innoche	Cat#D3850-500ML
TRIzol reagent	Ambion	Cat#15596018
PrimeScriptTM RT Master Mix	TaKaRa	Cat#RR036A
Power SYBR Green PCR Master Mix	Qiagen	Cat#208054
Glucose-6-phosphate	Sigma	G7879-500mg
DMEM	Gibco	11966–025
[U-^13^C_6_] glucose	CIL	CLM-1396-1
Nonlabeled glucose	Sigma	G7021

**Critical commercial assays**		

Widely targeted metabolomics assay	Wuhan Metware Biotechnology Co., Ltd	LC-ESI-MS/MS system analysis
Widely targeted lipidomics assay	Wuhan Metware Biotechnology Co., Ltd	LC-ESI-MS/MS system analysis
Metabolon-based energy metabolism detection	Applied Protein Technology, Shanghai, China	LC-MS/MS analysis

**Deposited data**		

Widely targeted metabolomics	This study	OMIX: OMIX003772
Widely targeted lipidomics assay	This study	OMIX: OMIX004117
Metabolon-based energy metabolism in PDAC organoids	This study	OMIX: OMIX004545
RNA expression data of PDAC organoids	This study	OMIX: OMIX003773
ATAC-seq peaks of PDAC organoids	This study	OMIX: OMIX003774
Coding mutations in PDAC organoids	This study	OMIX: OMIX003813

**Experimental models: Cell lines**		

HEK-293T	ATCC	Cat#CRL-1573

**Experimental models: Organisms/strains**		

Human PDAC organoids	This study	N/A
DH5α	AlpaLifeBio	KTSM101L

**Oligonucleotides**		

shRNA hairpins	This study	Table S6
qRT-PCR primers	This study	Table S6

**Software and algorithms**		

R version 4.0.3	The R Foundation for Statistical Computing	N/A
“CancerSubtypes” Bioconductor package for R	Xu et al.^71^	https://bioconductor.org/packages/release/bioc/html/CancerSubtypes.html
MBROLE 2.0	López-Ibáñez et al.^72^	https://csbg.cnb.csic.es/mbrole2/
“GSVA” Bioconductor package for R	Hänzelmann et al.^73^	https://www.bioconductor.org/packages/release/bioc/html/GSVA.html
“limma” Bioconductor package for R	Ritchie et al.^74^	https://bioconductor.org/packages/release/bioc/html/limma.html
Nearest Template Prediction (NTP)	Hoshida.^75^	https://github.com/genepattern/NearestTemplatePrediction/blob/master/src/NTPez.R
“clusterProfiler” Bioconductor package for R	Wu et al.^76^	https://bioconductor.org/packages/release/bioc/html/clusterProfiler.html
Bowtie v2.3.1	Langmead and Salzberg.^77^	https://bowtie-bio.sourceforge.net/bowtie2/index.shtml
Sambamba v0.6.6	Tarasov et al.^78^	https://lomereiter.github.io/sambamba/
Samtools v1.4	Li et al.^79^	https://samtools.sourceforge.net/
MACS2 v2.1.1	Zhang et al.^80^	https://hbctraining.github.io/Intro-to-ChIPseq/lessons/05_peak_calling_macs.html
deepTools	Ramírez et al.^81^	https://deeptools.readthedocs.io/en/develop/
“DiffBind” Bioconductor package for R	Ross-Innes et al.^82^	https://bioconductor.org/packages/release/bioc/html/DiffBind.html

### Resource availability

#### Lead contact

Further information and requests for resources and reagents should be directed to and will be fulfilled by the lead contact, Gang Jin (jingang@smmu.edu.cn).

#### Materials availability

This study did not generate new unique reagents.

### Experimental model and study participant details

#### Human subjects ethics statement

PDAC tissue samples were collected at Changhai Hospital. All patients involved in this study gave informed consent for the use of their clinical data and surgical specimens and agreed to the release of clinical information that could identify individuals. The protocols for this study were in conformity with national guidelines and received approval from Changhai Hospital’s ethics committee (approval no. CHEC2018-111). Furthermore, this study was approved by the Chinese Ministry of Science and Technology (MOST) for the Review and Approval of Human Genetic Resources (approval no. 2021BAT1264) in addition to the local IRB’s permission. A total of 28 patient cases from both male and female subjects between the ages of 40–79 years old were included in the present study. Detailed clinical information of these patients was listed in Table S1.

#### Organoid culture

PDAC tissues were digested in collagenase II (2.5 mg/mL with 10 μM Y-27632) at 37°C for approximately 30 min. The digested cells were washed with basic 1640 medium (10 μM Y-27632) for twice and centrifuged for 5min (1500rpm, RT). The obtained cells were embedded in Matrigel and overlaid with a previously described complete medium.^31^ The complete medium components are list in the key resource table. PDAC biopsy samples was directly cultured in complete medium without digestion. Organoids was cultured at 5% CO_2_ in 20% O_2_, and the media were changed every 4 days. The established organoids were for mycoplasma contamination weekly. All established PDAC organoids were expanded and stored as cryo-stocks. The media used for organoid cryopreservation were composed of the complete medium (90%) and 10% DMSO.

#### Mouse studies

Female SCID mice of 4-week-old were purchased from Biocytogen Pharmaceuticals (Beijing) Co., Ltd. All animal work was conducted in accordance with a protocol approved by the Institutional Animal Care and Use Committee (IACUC) of the Center for Excellence in Molecular Cell Science (CEMCS), and ethical approval was received from the IACUC of CEMCS. Mice were bred in specific pathogen free (SPF) animal house with 28°C and 50% humidity. Indicated cells were inoculated into mammary pad of the six-week-old female SCID mouse (n = 3 per group, 2 × 10^6^cells/injection). After the xenografts became palpable (∼200mm^3^), mice were injected with 5-FU (25 mg/kg, every 2 days), 6AN (5 mg/kg, every 2 days), MLN8054 (10 mg/kg, every 2 days), alisertib (10 mg/kg, every 2 days) intraperitoneal for about 2 weeks. Tumor size was measured every 2 days and the tumor volume was calculated with the equation *V* (in mm^3^) = 0.5 × length × width^2^. The animals were killed when the biggest xenografts near reached at ∼1500mm^3^.

### Method details

#### Widely targeted metabolomics assay

PDAC organoids in Matrigel (CORNING, 356231) were collected. Organoids were washed twice with 1mL of cold PBS buffer and were then centrifuged for 3 min at 500× g (4°C). The supernatant was removed and discarded; cell pallet was used for widely targeted metabolites assay (Wuhan Metware Biotechnology Co., Ltd). Sample was thawed on ice, then added 1 mL pre-cooled extractant (70% methanol aqueous solution), and whirl for 1 min. Freeze the mixture for 3 min in liquid nitrogen after remove ice for 3 min, it will be whirled for 2 min, circulate this at 3 times. Centrifuge the mixture again with 12000 r/min at 4°C for 10 min. Finally take the supernatant into the sample bottle for LC-ESI-MS/MS system analysis. In order to compare the substance content of all detected metabolites in different organoids, the chromatographic peaks detected in different samples for each metabolite were corrected to ensure qualitative and quantitative accurate according to the information of metabolite retention time and peak shape. Quality control samples are prepared by mixing sample extracts to test reproducibility.

#### Widely targeted lipidomics assay

Organoids were washed twice with 1mL of cold PBS buffer and were then centrifuged for 3 min at 500× g (4°C). The supernatant was removed and discarded; cell pallet was placed in liquid nitrogen for 2 min, then thawed on ice for 5 min and vortex blending. Repeat the first step 3 times, then centrifuge it with 12,000 rpm at 4°C for 10 min. Take 300 μL supernatant and homogenize it with 1mL mixture (include methanol, MTBE and internal standard mixture). Whirl the mixture for 2 min. Then add 500 μL of water and whirl the mixture for 1 min, and centrifuge it with 12,000 rpm at 4°C for 10 min. Extract 500 μL supernatant and concentrate it. Dissolve powder with 100 μL mobile phase B, then stored in −80°C. Finally take the dissolving solution into the sample bottle for LC-MS/MS analysis.

#### Metabolon-based energy metabolism detection

PDAC organoids in Matrigel were collected. Organoids were washed twice with 1mL of cold PBS buffer and were then centrifuged for 3 min at 500× g and 4°C. The supernatant was removed and discarded; cell pallet was used for metabolon-based energy metabolism detection (Applied Protein Technology, Shanghai, China). A homogenate of 100 mg of sample mixed with 1 mL of cold methanol/acetonitrile/H_2_O (2:2:1, v/v/v) was sonicated at a low temperature (30 min/once, twice) and then centrifuged for 20min (140,00g, 4°C). The supernatant was dried in a vacuum centrifuge. For LC-MS analysis, the dried samples were dissolved in 100 μL acetonitrile/water (1:1, v/v), adequately vortexed and then centrifuged (140,00 rpm, 4°C, 15 min). The supernatants were collected for the LC-MS/MS analysis. Analyses were performed using an UHPLC (1290 Infinity LC, Agilent Technologies) coupled to a QTRAP (AB Sciex 5500).

#### Metabolic flux experiments using [U-^13^C_6_] glucose

Organoids were seeded at a density of approximately 1×10^7^ cells per 10 cm dish. The labeling medium composed of DMEM (Gibco, 11966-025) with a supplement of 1 g/L [U-^13^C_6_] glucose (CIL, CLM-1396-1), 1 g/L nonlabeled glucose (Sigma, G7021), 10% (v/v) FBS, 1mM pyruvate, unlabeled 2mM L-glutamine and 1% (v/v) penicillin-streptomycin. The labeling time of [U-^13^C_6_] glucose was 24h. Metabolic flux experiments were performed according to a previous report.^41^^,^^83^

#### Measurement of labeled metabolites of isotopomers by GC-MS, LC-MS and UHPLC-QTOF system

Metabolites of glycolysis, TCA cycle, oxidative PPP and nonoxidative PPP were measured followed a previously published protocol.^41^^,^^83^

#### Measurement of G6PD enzymatic activity

G6PD activity was measured at room temperature. G6PD was immunoprecipitated from the lysates of organoids and subjected to G6PD enzymatic activity assays in the reaction buffer containing 42 mM Tris (PH 7.5), 2.66mM Glucose-6-phosphate (Sigma, G7879-500mg), 40mM MgCl_2_, 0.66 mM β-NADP. The change in absorbance at 340 nM owing to increase of NADPH was measured using BioTek Synergy *Neo* Multi-Mode Plate Reader (BioTek, USA).

#### Immunoprecipitation and immunoblotting

For pancreatic cancer organoid cells, cell lysate (25 mM Tris-HCl pH 8.0, 150 mM NaCl, 1 mM CaCl2, 1%, 1 Triton X-100) with EDTA-free protease inhibitors (Biotool) was added to the dish, gently scraped with cell scraping and transferred to a 1.5 mL centrifuge tube, sonicated for 2s, 0.5s apart with a total length of 0.3 min, and let for 30 min on ice for full lysis. Treated cell homogenates at 4°C were centrifuged at 12,000 r p m for 10 min. Protein concentration was detected using protein kit. The Protein A/G PLUS-beads (Santa Cruz) was closed in 4°C with 0.1% BSA for 1 h, washed three times with Wash buffer, centrifuged at 400 g to remove the waste liquid, and the beads was stored in a 4°C refrigerator. After the protein concentration was determined, the cell lysate was quantified to a total protein amount of 2 mg, and the primary antibody was added to the cell lysate at a ratio of 1:100, incubated overnight at 4°C to bind the antibody to the target protein. The next day, the mixture of antibody and protein was incubated with the blocked Protein A/G PLUS-Beads for 6 h at 4°C to bind the immunoprecipitated complex to beads. By centrifugation at 300*g* of the cells for 4 min at 4°C, washing the beads with Wash buffer (50 mM Tris-HCl, 400 Mm NaCl and 0.8% Triton X-100, pH 7.5) for 3–5 min each. After the second wash, it was transferred to a new 1.5 mL centrifuge tube and then repeated twice, finally adding 90 μL prediluted 1X loading buffer, 95°C metal bath for 10 min and 12,000 r p m for 3 min to dissociate the protein from the beads for western assay.

#### Drug-treatment assays

384-well plates (corning, 3765) were coated with 10 μL of collagen before the addition of PDAC organoids (3,000 cells per well, 50 μL). Chemotherapeutic agents as well as DMSO (Innoche, D3850-500ML) controls were added in triplicate using HP D300e Digital Dispenser. Cell viability was assayed by CellTiter-Glo Reagent (Promega, G7573), SRB solution (Sigma, S1403-25g) or CCK-8 (Vazyme, A311-01) after five days treatment. To measure sensitivity, we used 5-8-point dose-response curves; for each drug, the corresponding cell viability values were used as input for curve generation. Average inhibition rates from three independent experiments were calculated with Excel and visualized using GraphPad Prism 8. Each drug concentration (nM/L) was log10 transformed. The AUC was calculated with the sintegral function in R, and the normalized AUC was obtained by dividing one AUC by the maximum AUC for each drug. GEM (TargetMol, T0251); 5-FU (SIGMA, F6627); IRI (Selleck chemicals, S1198); OXA (Selleck chemicals, S1124); PTX (Selleck chemicals, S1150); BAY-876 (Selleck chemicals, S8452); 6AN (Selleck chemicals, S9783); MLN8054 (TargetMol; T6315); Alisertib (Selleck chemicals, S1133); Pyrimidine nucleosides include uridine (Sigma, U3003), cytidine (Sigma, C4654), thymidine (Sigma, T1895), with a final concentration of 240um; Purine nucleosides include Guanosine (Sigma, G6264) and adenosine (Sigma, A4036), with a final concentration of 200um; R5P (MCE, 207671-46-3) with a final concentration of 1mM.

#### Immunohistochemistry

Immunohistochemistry was performed as described in our previous study.^31^ The antibodies used for staining TMA was as follows: Anti-GLUT1 (1/500, abcam, ab115730) and anti-ALDOB (1/2000, proteintech, 18065-1-AP).

#### qRT-PCR

Total RNA was extracted with TRIzol reagent (Ambion, 15596018) from cells for the generation of single stranded cDNA. Reverse transcription was further performed with PrimeScriptTM RT Master Mix (TaKaRa, RR036A) with 500 ng of total RNA as input. Quantitative RT-PCR (qRT-PCR) was performed using an ABI 7300 Real-Time PCR System (Applied Biosystems) with the Power SYBR Green PCR Master Mix (Qiagen, 208054). The primers used for each of the genes are listed (Table S6).

#### Metabolomics-based subtyping

Unsupervised classification of PDAC metabolomics was conducted with consensus clustering (R package “CancerSubtypes”; clusterAlg = "hc", distance = "euclidean", innerLinkage = "ward.D2").^71^ Differential compounds in each subtype were calculated by Wilcoxon rank-sum test (P-value<0.05, Fold-change>1.2) and functional enrichment analysis was performed by MBROLE 2.0 with dysregulated metabolomics (FDR<0.05).^72^ Single-sample gene set enrichment analysis (ssGSEA) was used to calculate enrichment score of the six metabolic ontology classes in two metabolic subtypes (R package “GSVA”).^73^

Differential expressed genes (DEGs) of two metabolic subtypes were calculated by R package limma with corresponding RNA-seq data (log2 transformed FPKM values, cutoff: P-value<0.05 and Fold-change>1.5).^74^ We then used these signature genes to make class prediction of Bailey PDAC cohort and TCGA PDAC cohort by NearestTemplatePrediction (NTP) and split these two cohorts into glucomet and lipomet subtype respectively.^75^ Only Squamous and Pancreatic progenitor samples for Bailey cohort (n = 55) and ductal pancreatic cancers samples were included for TCGA cohort (n = 156). In addition, gene set enrichment analysis (GSEA) was performed to determine KEGG pathways enriched in two metabolic subtypes with ranked genes list (R package “clusterProfiler”).^76^^,^^84^

Corresponding whole-genome sequencing data were also used to explore genomic difference between two metabolic subtypes. Nonsynonymous somatic mutations were counted and then divided by the size of the coding region (∼45M) to calculate tumor mutation burden (TMB). To calculate the chromosome instability (CIN), we used a weighted-sum approach following another study.^85^ First, absolute log2 ratios of all CNV segments within a chromosome were weighted by the segment length and summed up to derive the instability score for each chromosome. Then, the genome-wide chromosome instability index was obtained by summing up the instability score of all 22 autosomes. Wilcoxon rank-sum test was used to calculate difference of TMB and CIN between two metabolic subtypes.

#### ATAC-data processing

Raw fastq data of ATAC-seq was mapped to the human reference genome hg19 with Bowtie v2.3.1 and then duplicate reads were removed with Sambamba v0.6.6.^77^^,^^78^ Then Samtools v1.4 was used to filter uniquely aligned reads and reads of chrM were filtered out.^79^ Peak calling was conducted with MACS2 v2.1.1 and the threshold was set as p < 0.0001.^80^ R package DiffBind was then used to compute differential peaks among different conditions (p < 0.05).^82^ In addition, deepTools bamCoverage (with parameters –normalizeUsingRPKM) was used to converted bam files into bigwig format and then deepTools plotHeatmap was used for visualization of differential peaks.^81^

#### Crystal structure of G6PD and substrate

The crystal structure of the complex of human G6PD with substrate glucose 6-phosphate(G6P) (PDB ID: 2BHL)^86^ was aligned to the complex of human G6PD with the structural NADP and coenzyme NADP (PDB ID: 2BH9), after being prepared by Protein Preparation Wizard (Schrödinger Release 2022-1: Protein Preparation Wizard; Epik, Schrödinger, LLC, New York, NY, 2022; Impact, Schrödinger, LLC, New York, NY; Prime, Schrödinger, LLC, New York, NY, 2022). Then G6P in 2BHL was extracted and merged with 2BH9. A conformation minimization of the amino acids in the merged structure around G6P (within 5 Å) was carried out by Prime (Schrödinger Release 2022-1: Prime, Schrödinger, LLC, New York, NY, 2022). The minimized complex was taken as the receptor for virtual screening with G6P binding site as the grid center. The compounds (7098 compounds) were prepared by LigPrep (Schrödinger Release 2022-1: LigPrep, Schrödinger, LLC, New York, NY, 2022) and docked into the receptor at the SP precision by Glide (Schrödinger Release 2022-1: Glide, Schrödinger, LLC, New York, NY, 2022). The docked ligand-protein complexes in 3D and the 2D ligand-protein interaction diagrams were presented by Maestro (Schrödinger Release 2022-1: Maestro, Schrödinger, LLC, New York, NY, 2022).

### Quantification and statistical analysis

Experimental data were analyzed by Student’s *t* test, Wilcoxon rank-sum test, Pearson correlation analysis or log rank test. The detailed statistical tests were indicated in figures legends.

## Data Availability

The data reported in this paper have been deposited in the OMIX, China National Center for Bioinformation/Beijing Institute of Genomics, Chinese Academy of Sciences (https://ngdc.cncb.ac.cn/omix: accession no.OMIX003772, no.OMIX003773, no.OMIX003774, no.OMIX003813, no.OMIX004117, no.OMIX004545). This paper does not report original code. Any additional information required to reanalyze the data reported in this work paper is available from the lead contact upon request.

## Supplemental information

Document S1. Figures S1–S6

Table S1. Clinicopathologic variables of 28 patient-derived PDAC organoids, related to Figure 1

Table S2. Differential metabolites of the two metabolic subtypes, related to Figure 1

Table S3. Glucomet-PDAC and lipomet-PDAC signature genes, related to Figure 1

Table S4. Normalized AUC of drug sensitivity in PDAC organoids with 4 chemotherapeutic drugs, related to Figure 2

Table S5. Tissue microarray (TMA) analyses on GLUT1 and ALDOB protein expressions, related to Figure 4

Table S6. The primers used for each of the genes, related to Figures S2 and S5

Document S2. Article plus supplemental information
