# Supplementary material for: Metabolic classification suggests the GLUT1/ALDOB/G6PD axis as a therapeutic target in chemotherapy-resistant pancreatic cancer
Source: Cell Rep Med. 2023 Aug 18;4(9):101162. doi: 10.1016/j.xcrm.2023.101162 (PMC10518604; doi:10.1016/j.xcrm.2023.101162)
Supplement: Document S1. Figures S1–S6 [file mmc1.pdf]

Cell Reports Medicine, Volume *4*

Supplemental information

Metabolic classification suggests the GLUT1/ALDOB/G6PD axis as a therapeutic target in chemotherapy-resistant pancreatic cancer

Yunguang Li, Shijie Tang, Xiaohan Shi, Jingwen Lv, Xueyuan Wu, Yehan Zhang, Huan Wang, Juan He, Yiqin Zhu, Yi Ju, Yajuan Zhang, Shiwei Guo, Weiwei Yang, Huiyong Yin, Luonan Chen, Dong Gao, and Gang Jin

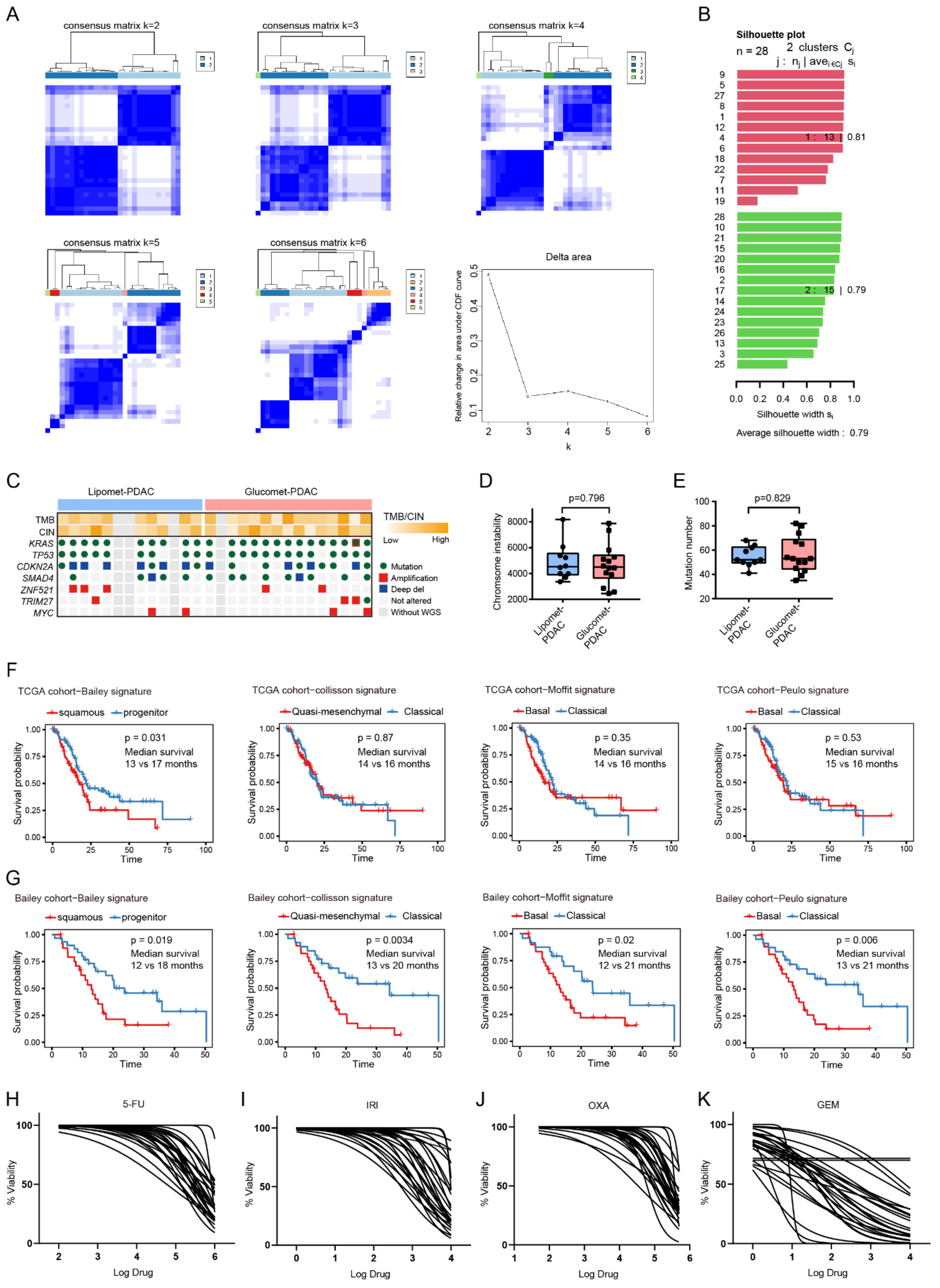
A
consensus matrix k=2
consensus matrix k=3
consensus matrix k=4
consensus matrix k=5
consensus matrix k=6
Delta area
Relative change in area under CDF curve
k
B
Silhouette plot
n = 28
2 clusters C_j
j : n_j | $ave_{i \in Cj}$ s_i
1 : 13 | 0.81
2 : 15 | 0.79
Silhouette width s_i
Average silhouette width : 0.79
C
Lipomet-PDAC
Glucomet-PDAC
TMB
CIN
KRAS
TP53
CDKN2A
SMAD4
ZNF521
TRIM27
MYC
TMB/CIN
Low
High
Mutation
Amplification
Deep del
Not altered
Without WGS
D
p=0.796
Chromsome instability
Lipomet-PDAC
Glucomet-PDAC
E
p=0.829
Mutation number
Lipomet-PDAC
Glucomet-PDAC
F
TCGA cohort−Bailey signature
squamous
progenitor
p = 0.031
Median survival
13 vs 17 months
TCGA cohort−collison signature
Quasi-mesenchymal
Classical
p = 0.87
Median survival
14 vs 16 months
TCGA cohort−Moffit signature
Basal
Classical
p = 0.35
Median survival
14 vs 16 months
TCGA cohort−Peulo signature
Basal
Classical
p = 0.53
Median survival
15 vs 16 months
Survival probability
Time
G
Bailey cohort−Bailey signature
squamous
progenitor
p = 0.019
Median survival
12 vs 18 months
Bailey cohort−collisson signature
Quasi-mesenchymal
Classical
p = 0.0034
Median survival
13 vs 20 months
Bailey cohort−Moffit signature
Basal
Classical
p = 0.02
Median survival
12 vs 21 months
Bailey cohort−Peulo signature
Basal
Classical
p = 0.006
Median survival
13 vs 21 months
Survival probability
Time
H
5-FU
I
IRI
J
OXA
K
GEM
% Viability
Log Drug

Figure S1. Metabolic profiles divide PDAC into two subtypes. Related to Figure 1 and Figure 2. (A) Unsupervised classification of PDAC metabolomics using consensus clustering. Solutions are shown for k = 2 to k = 6 classes. A peak cophenetic correlation is observed for k = 2 classes. (B) Silhouette information for k = 2 classes. (C) Analysis of alterations at the genomic level among the two metabolic subgroups. The gene alterations are indicated as follows: Top cancer-related genes with protein-coding mutations (green); Top cancer-related copy number alteration status is amplification (red) and deep deletion (blue); wild-type (light gray). (D and E) Comparison of chromosome instability (D) and mutation number (E) between glucomet-PDAC and lipomet-PDAC. The boxplot shows the median (central line) and the 25-75% interquartile range (box limits). (F and G) Kaplan-Meier survival curves of the TCGA PDAC cohort (F) and Bailey PDAC cohort (G) split independently by RNA signatures (Bailey signature, Collisson signature, Moffit signature and Peulo signature). (H-K) Dose-response curves and normalized AUC distribution for 5-FU (H), IRI (I), OXA (J), and GEM (K) on glucomet-PDAC and lipomet-PDAC. Statistical significance was computed by unpaired Student's t test (D and E). Statistical significance was computed by log-rank test (F and G).

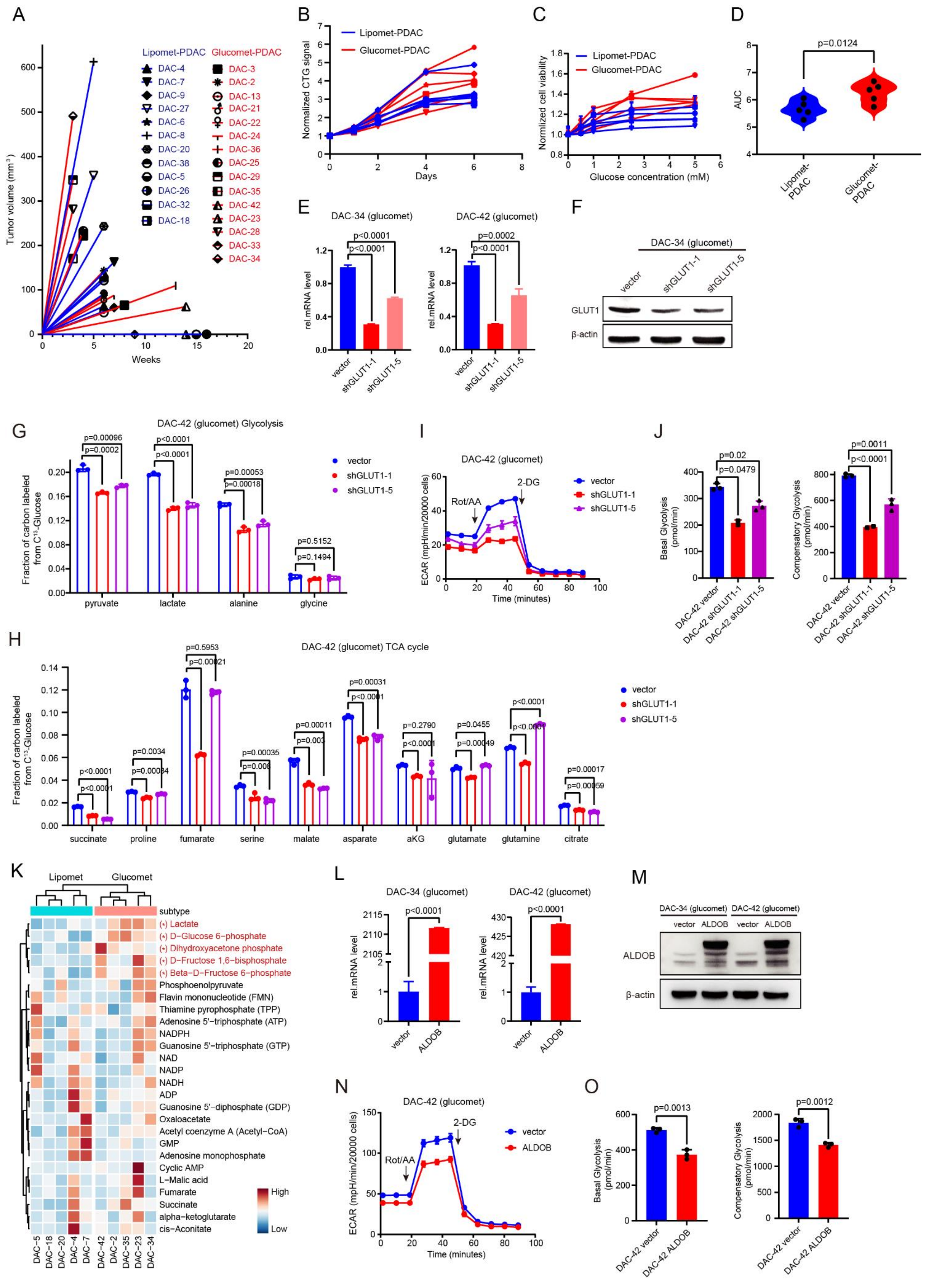
A
Lipomet-PDAC
Glucomet-PDAC
DAC-4
DAC-7
DAC-9
DAC-27
DAC-6
DAC-8
DAC-20
DAC-38
DAC-5
DAC-26
DAC-32
DAC-18
DAC-3
DAC-2
DAC-13
DAC-21
DAC-22
DAC-24
DAC-36
DAC-25
DAC-29
DAC-35
DAC-42
DAC-23
DAC-28
DAC-33
DAC-34
Tumor volume (mm³)
Weeks
B
Lipomet-PDAC
Glucomet-PDAC
Normalized CTG signal
Days
C
Lipomet-PDAC
Glucomet-PDAC
Normlized cell viability
Glucose concentration (mM)
D
p=0.0124
AUC
Lipomet-PDAC
Glucomet-PDAC
E
DAC-34 (glucomet)
p<0.0001
p<0.0001
DAC-42 (glucomet)
p=0.0002
p<0.0001
rel.mRNA level
vector
shGLUT1-1
shGLUT1-5
F
DAC-34 (glucomet)
vector
shGLUT1-1
shGLUT1-5
GLUT1
β-actin
G
DAC-42 (glucomet) Glycolysis
p=0.00096
p=0.0002
p<0.0001
p<0.0001
p=0.00053
p=0.00018
p=0.5152
p=0.1494
Fraction of carbon labeled from C¹³-Glucose
pyruvate
lactate
alanine
glycine
vector
shGLUT1-1
shGLUT1-5
I
DAC-42 (glucomet)
Rot/AA
2-DG
vector
shGLUT1-1
shGLUT1-5
ECAR (mpH/min/20000 cells)
Time (minutes)
J
p=0.02
p=0.0479
Basal Glycolysis (pmol/min)
p=0.0011
p<0.0001
Compensatory Glycolysis (pmol/min)
DAC-42 vector
DAC-42 shGLUT1-1
DAC-42 shGLUT1-5
H
DAC-42 (glucomet) TCA cycle
p<0.0001
p<0.0001
p=0.0034
p=0.00084
p=0.5953
p=0.00021
p=0.00035
p=0.008
p=0.00011
p=0.003
p=0.00031
p<0.0001
p=0.2790
p<0.0001
p=0.0455
p=0.00049
p<0.0001
p<0.0001
p=0.00017
p=0.00059
Fraction of carbon labeled from C¹³-Glucose
succinate
proline
fumarate
serine
malate
asparate
aKG
glutamate
glutamine
citrate
vector
shGLUT1-1
shGLUT1-5
K
Lipomet
Glucomet
subtype
(+) Lactate
(+) D-Glucose 6-phosphate
(+) Dihydroxyacetone phosphate
(+) D-Fructose 1,6-bisphosphate
(+) Beta-D-Fructose 6-phosphate
Phosphoenolpyruvate
Flavin mononucleotide (FMN)
Thiamine pyrophosphate (TPP)
Adenosine 5'-triphosphate (ATP)
NADPH
Guanosine 5'-triphosphate (GTP)
NAD
NADP
NADH
ADP
Guanosine 5'-diphosphate (GDP)
Oxaloacetate
Acetyl coenzyme A (Acetyl-CoA)
GMP
Adenosine monophosphate
Cyclic AMP
L-Malic acid
Fumarate
Succinate
alpha-ketoglutarate
cis-Aconitate
High
Low
DAC-5
DAC-18
DAC-20
DAC-4
DAC-7
DAC-42
DAC-2
DAC-35
DAC-23
DAC-34
L
DAC-34 (glucomet)
p<0.0001
DAC-42 (glucomet)
p<0.0001
rel.mRNA level
vector
ALDOB
M
DAC-34 (glucomet)
DAC-42 (glucomet)
vector
ALDOB
vector
ALDOB
ALDOB
β-actin
N
DAC-42 (glucomet)
Rot/AA
2-DG
vector
ALDOB
ECAR (mpH/min/20000 cells)
Time (minutes)
O
p=0.0013
Basal Glycolysis (pmol/min)
p=0.0012
Compensatory Glycolysis (pmol/min)
DAC-42 vector
DAC-42 ALDOB

Figure S2. GLUT1 and ALDOB drive glycolysis and TCA cycle reprogramming in glucomet-PDAC. Related to Figure 3. (A) Tumor growth curves were analyzed in a xenograft mouse model injected with the indicated organoids. The slope of the curve represents the growth rate of the xenografts. (B) The proliferative rates of represented organoids of two metabolic subtypes in vitro (n = 3 technical replicates). ATP content in cells was detected on days 0, 1, 2, 4 and 6 by CellTiter-Glo assays. (C) CellTiter-Glo assays performed on equal numbers of growtharrested cells from glucomet-PDAC show an elevated signal compared to lipomet-PDAC (n = 3 technical replicates). (D) Normalized AUC distribution for the glucose effect on organoid growth. The significance of the difference was determined by unpaired Student's t test. (E) RT-PCR analysis *GLUT1* knockdown efficiency in the indicated organoids (n = 3 technical replicates). (F) Western blot analysis of GLUT1 knockdown efficiency in DAC-34 (glucomet) organoids. (G and H) Fractions of labeled metabolites in glycolysis (G) and the TCA cycle (H) from [U-$^{13}C_6$] glucose in control and *GLUT1* knockdown organoids (n = 3 technical replicates). (I and J) Control and *GLUT1* knockdown organoids (DAC-42) were exposed to rotenone/antimycin A and 2-DG to measure the ECAR at the basal level and compensatory level by the Seahorse XF Glycolytic Rate Assay (n = 3 technical replicates). (K) Heatmap of Metabolon-based energy metabolism showing the metabolite intensity differences in glucomet-PDAC and lipomet-PDAC. Red denotes significant intensity differences in metabolites. Significance was computed by the Wilcoxon rank-sum test (*: $p<0.05$). (L) RT-PCR analysis of *ALDOB* overexpression efficiency in the indicated organoids (n = 3 technical replicates). (M) Western blot analysis of ALDOB overexpression in DAC-34 (glucomet) and DAC-42 (glucomet) organoids. (N and O) Control and *ALDOB* overexpression organoids (DAC-42) were exposed to rotenone/antimycin A and 2-DG to measure the ECAR at the basal level and compensatory level by the Seahorse XF Glycolytic Rate Assay (n = 3 technical replicates). Data are presented as the mean values ± SEMs, statistical significance was computed by unpaired Student's t test.

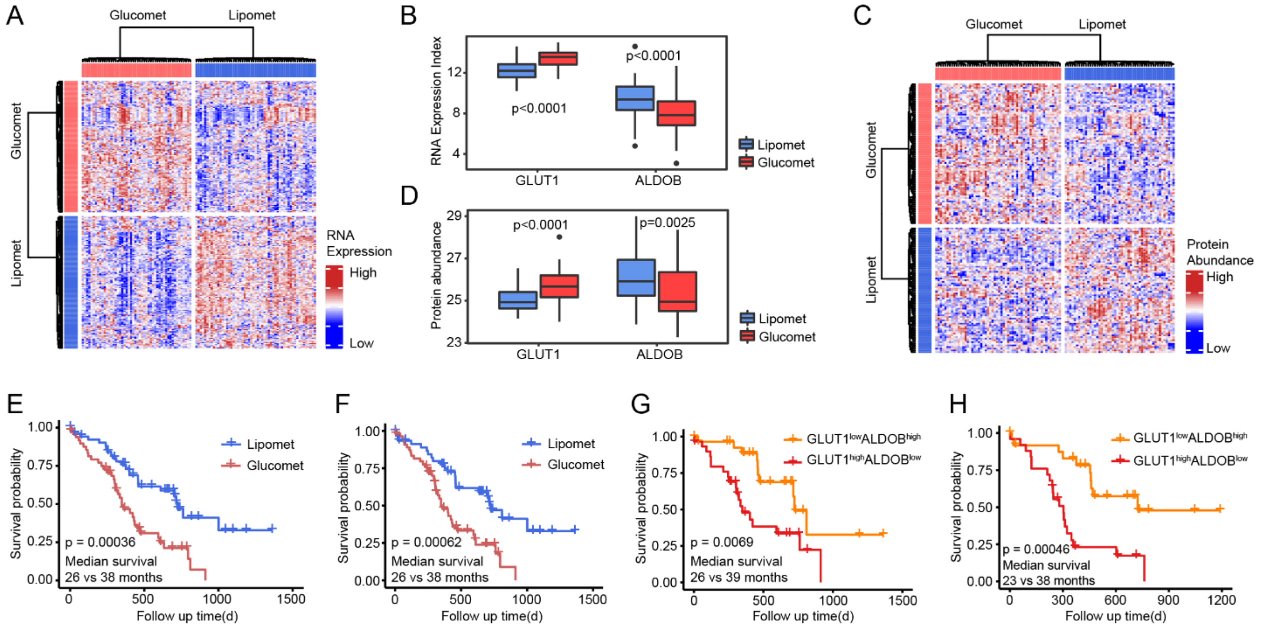

Figure S3. Consensus-clustered of tumors in the published PDAC cohort split by metabolism signature genes. Related to Figure 4. (A) Heatmap of tumors in the published PDAC cohort (n = 105) split by glucomet and lipomet signature genes in RNA level. (B) Boxplot of *GLUT1* and *ALDOB* RNA expression levels in the PDAC cohort stratified by metabolic subgroup. (C) Heatmap of tumors in the PDAC cohort (n = 105) split by glucomet and lipomet signature genes in protein level. (D) Boxplot of GLUT1 and ALDOB protein expression levels in the PDAC cohort stratified by metabolic subgroup. (E and F) Kaplan-Meier survival curves of the Cao PDAC cohort showing differential prognosis among patients with different RNA (E) and protein (F) subtypes. (G and H) Kaplan-Meier survival curves based on the expression of *GLUT1* and *ALDOB* in RNA (G) and protein (H) levels. Data are presented as the mean values ± SEMs, statistical significance was computed by limma (B and D). Statistical significance was computed by log-rank test (E-H).

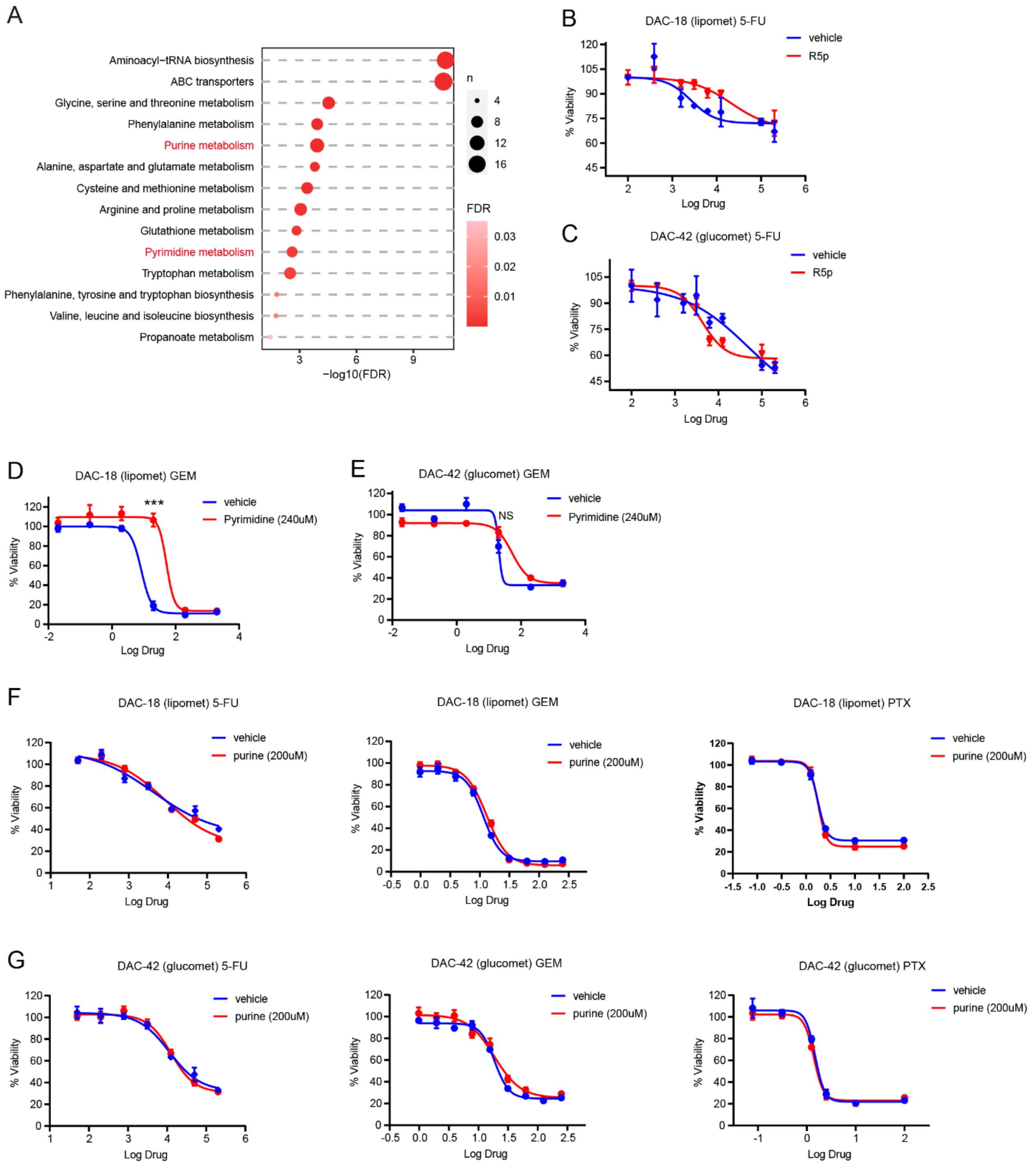

Figure S4. Increased nucleoside synthesis in glucomet-PDAC. Related to Figure 5. (A) Enrichment analysis of elevated metabolomics in glucomet subtypes (n = 140 metabolomics, FDR < 0.05, MBROLE 2.0). (B and C) Effect of R5P (1 mM) on 5-FU sensitivity in DAC-18 (lipomet) and DAC-42 (glucomet) cells by CellTiter-Glo assays at 5 days posttreatment. (D and E) DAC-18 (lipomet) and DAC-42 (glucomet) organoids were treated with GEM alone or in combination with pyrimidine nucleosides (uridine, cytidine and thymidine, 240µM), and cell viability was determined by CellTiter-Glo assays. (F and G) DAC-18 (lipomet) and DAC-42 (glucomet) organoids were treated with the indicated chemo alone or in combination with purine nucleosides (guanosine and adenosine, 200 µM) for 5 days, and cell viability was determined by CellTiter-Glo assays. All dose-responsive curves are performed with 3 technical replicates. Data are presented as the mean values ± SEMs, statistical significance was computed by unpaired Student's t test (*: $p<0.05$; **: $p<0.01$; ***: $p<0.001$).

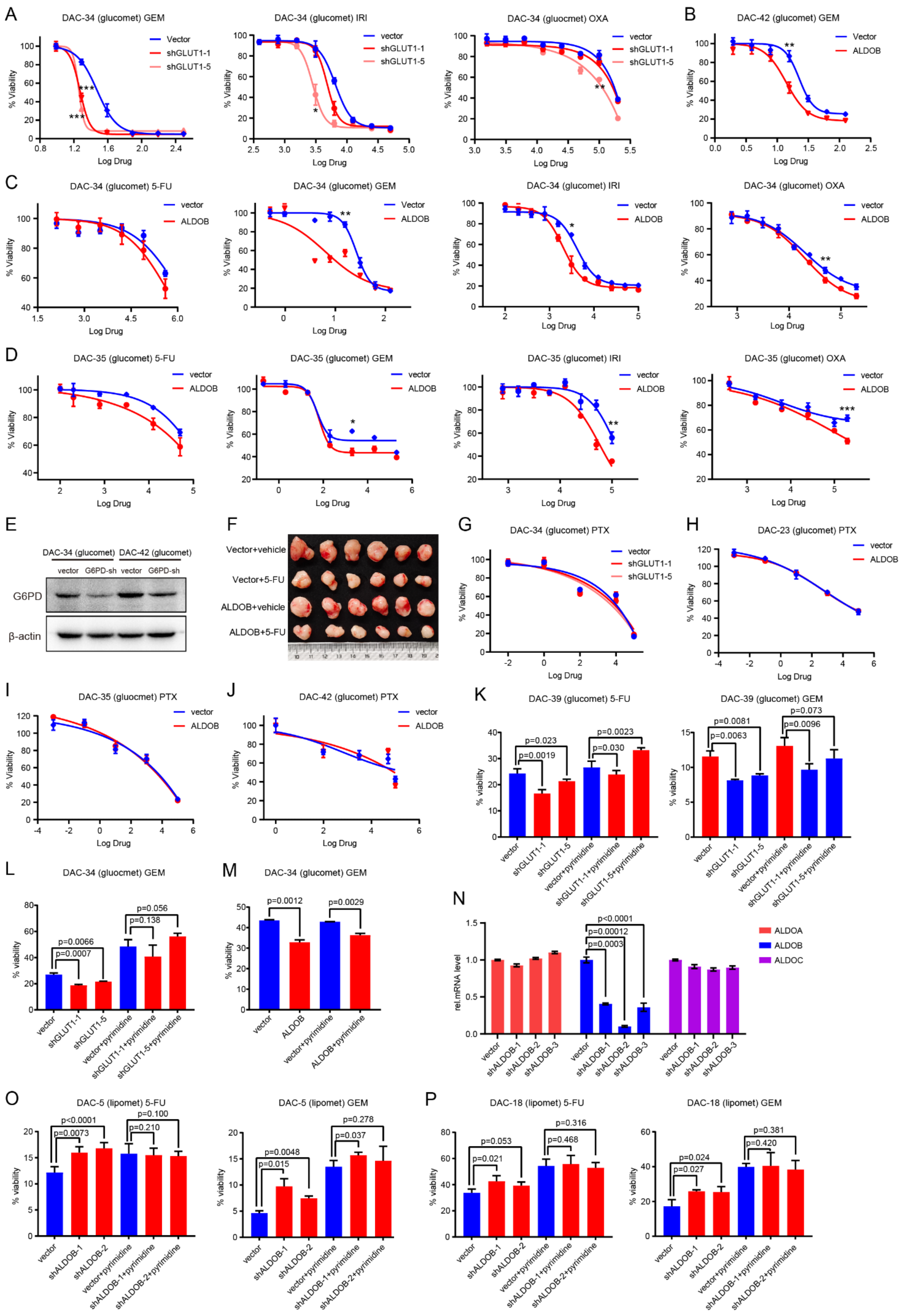
A
DAC-34 (glucomet) GEM
DAC-34 (glucomet) IRI
DAC-34 (gluocmet) OXA
Vector
shGLUT1-1
shGLUT1-5
% Viability
Log Drug
B
DAC-42 (glucomet) GEM
ALDOB
C
DAC-34 (glucomet) 5-FU
DAC-34 (glucomet) GEM
DAC-34 (glucomet) IRI
DAC-34 (glucomet) OXA
D
DAC-35 (glucomet) 5-FU
DAC-35 (glucomet) GEM
DAC-35 (glucomet) IRI
DAC-35 (glucomet) OXA
E
DAC-34 (glucomet)
DAC-42 (glucomet)
vector
G6PD-sh
G6PD
β-actin
F
Vector+vehicle
Vector+5-FU
ALDOB+vehicle
ALDOB+5-FU
G
DAC-34 (glucomet) PTX
H
DAC-23 (glucomet) PTX
I
DAC-35 (gluocmet) PTX
J
DAC-42 (glucomet) PTX
K
DAC-39 (glucomet) 5-FU
DAC-39 (glucomet) GEM
% viability
vector
shGLUT1-1
shGLUT1-5
vector+pyrimidine
shGLUT1-1+pyrimidine
shGLUT1-5+pyrimidine
p=0.023
p=0.0019
p=0.0023
p=0.030
p=0.0081
p=0.0063
p=0.073
p=0.0096
L
DAC-34 (gluocmet) GEM
p=0.0066
p=0.0007
p=0.056
p=0.138
M
DAC-34 (glucomet) GEM
ALDOB
ALDOB+pyrimidine
p=0.0012
p=0.0029
N
rel.mRNA level
ALDOA
ALDOB
ALDOC
shALDOB-1
shALDOB-2
shALDOB-3
p<0.0001
p=0.00012
p=0.0003
O
DAC-5 (lipomet) 5-FU
DAC-5 (lipomet) GEM
shALDOB-1+pyrimidine
shALDOB-2+pyrimidine
p<0.0001
p=0.0073
p=0.100
p=0.210
p=0.0048
p=0.015
p=0.278
p=0.037
P
DAC-18 (lipomet) 5-FU
DAC-18 (lipomet) GEM
p=0.053
p=0.021
p=0.316
p=0.468
p=0.024
p=0.027
p=0.381
p=0.420

Figure S5. The GLUT1/ALDOB/G6PD axis contributes to drug resistance. Related to Figure 5. (A) Effect of *GLUT1* knockdown on GEM, IRI and OXA responsiveness of DAC-34 (glucomet) as determined by CellTiter-Glo assays 120 hr after treatment with GEM, IRI or OXA. (B-D) Effect of *ALDOB* overexpression on the response to the indicated chemotherapy for DAC-42 (glucomet), DAC-34 (glucomet) and DAC-35 (glucomet) as determined by CellTiter-Glo assays 120 hr after treatment with chemotherapy. (E) Western blot analysis of G6PD knockdown efficiency in DAC-34 (glucomet) and DAC-42 (glucomet) organoids. (F) Image of xenografts in the ODX model isolated from each group at day 16 of drug treatment. (G) Effect of *GLUT1* knockdown on PTX of DAC-34 (glucomet) as determined by CellTiter-Glo assays. (H-J) Effect of *ALDOB* overexpression on PTX of DAC-23 (glucomet), DAC-35 (glucomet) and DAC-42 (glucomet) as determined by CellTiter-Glo assays 120 hr after treatment with chemotherapy. (K and L) Effect of pyrimidine nucleotide (240 μM) on 5-FU or GEM sensitivity in control and *GLUT1* knockdown organoids by CellTiter-Glo assays at 120 hr posttreatment. (M) Effect of pyrimidine nucleotide (240 μM) on GEM sensitivity in control and *ALDOB*-overexpressing organoids by CellTiter-Glo assays at 120 hr posttreatment. (N) RT-PCR analysis of *ALDOB* knockdown efficiency and specificity in DAC-5 (lipomet) organoids. (O and P) Effect of pyrimidine nucleotides (240 μM) on 5-FU and GEM sensitivity in control and *ALDOB* knockdown DAC-5 (lipomet) or DAC-18 (lipomet) organoids by CellTiter-Glo assays at 120 hr posttreatment. All dose-responsive curves are performed with 3 technical replicates. All dose-responsive curves are performed with 3 technical replicates. Data are presented as the mean values ± SEMs, statistical significance was computed by unpaired Student's t test (*: $p<0.05$; **: $p<0.01$; ***: $p<0.001$) (A-D).

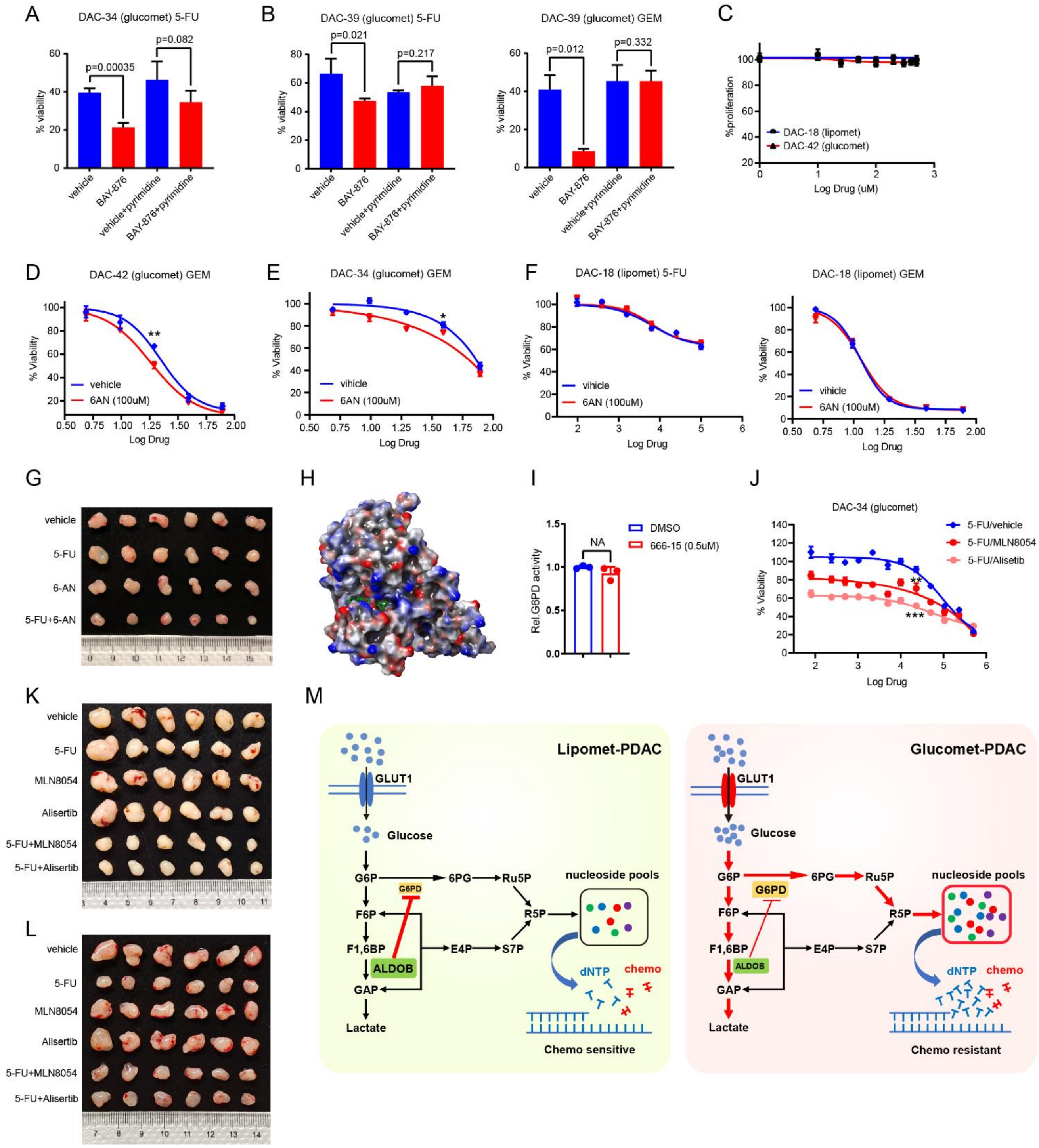

Figure S6. Pharmacological inhibition of GLUT1 or G6PD improves chemotherapy sensitivity. Related to Figure 6. (A and B) Effect of pyrimidine nucleotides (240 μM) on 5-FU and GEM sensitivity in control and GLUT1 inhibition glucomet-PDAC (DAC-34 and DAC-39) by CellTiter-Glo assays at 120 hr posttreatment (n = 3 technical replicates). (C) The effect of the G6PD inhibitor 6AN on cell viability was evaluated in DAC-18 (lipomet) and DAC-42 (glucomet) organoids (n = 3 technical replicates). (D and E) Effect of 6AN treatment on GEM responsiveness of DAC-42 (D) and DAC-34 (E) organoids as determined by CellTiter-Glo assays 120 hr after treatment with GEM. (F) Effect of 6AN treatment on 5-FU and GEM responsiveness of DAC-18 (lipomet) organoids as determined by CellTiter-Glo assays 120 hr after treatment with 5-FU or GEM. (G) Image of DAC-42 (glucomet) xenografts in the ODX model isolated from each group at day 8 of drug treatment (n = 6 tumors). (H) Prediction of the potential interaction sites in the 3D structure of the G6PD

protein with 666-15. (I) Effect of 666-15 on G6PD activity in DAC-42 (glucomet) organoids (n = 3 technical replicates). (J) Effect of MLN8054 or alisertib treatment on the 5-FU responsiveness of DAC-34 (glucomet) organoids as determined by CellTiter-Glo assays 120 hr after treatment with 5-FU. (K and L) Image of glucomet-PDAC DAC-42 (K) and lipomet-PDAC DAC-18 (L) xenografts in the ODX model isolated from each group with indicated drug treatment (n = 6 tumors). (M) Schematic model for glucose reprogramming explaining the glucomet-PDAC drug resistance mechanism. All dose-responsive curves are performed with 3 technical replicates. Data are presented as the mean values ± SEMs, statistical significance was computed by unpaired Student's t test (*: $p<0.05$; **: $p<0.01$; ***: $p<0.001$) (A-F, I and J).
